# Use of Multiprognostic Index Domain Scores, Clinical Data, and Machine Learning to Improve 12-Month Mortality Risk Prediction in Older Hospitalized Patients: Prospective Cohort Study

**DOI:** 10.2196/26139

**Published:** 2021-06-21

**Authors:** Richard John Woodman, Kimberley Bryant, Michael J Sorich, Alberto Pilotto, Arduino Aleksander Mangoni

**Affiliations:** 1 College of Medicine and Public Health Flinders University Adelaide Australia; 2 Department of Geriatric Care OrthoGeriatrics and Rehabilitation Galliera Hospital Genoa Italy; 3 Department of Interdisciplinary Medicine University of Bari Bari Italy

**Keywords:** machine learning, Multidimensional Prognostic Index, mortality, diagnostic accuracy, XGBoost

## Abstract

**Background:**

The Multidimensional Prognostic Index (MPI) is an aggregate, comprehensive, geriatric assessment scoring system derived from eight domains that predict adverse outcomes, including 12-month mortality. However, the prediction accuracy of using the three MPI categories (mild, moderate, and severe risk) was relatively poor in a study of older hospitalized Australian patients. Prediction modeling using the component domains of the MPI together with additional clinical features and machine learning (ML) algorithms might improve prediction accuracy.

**Objective:**

This study aims to assess whether the accuracy of prediction for 12-month mortality using logistic regression with maximum likelihood estimation (LR-MLE) with the 3-category MPI together with age and gender (feature set 1) can be improved with the addition of 10 clinical features (sodium, hemoglobin, albumin, creatinine, urea, urea-to-creatinine ratio, estimated glomerular filtration rate, C-reactive protein, BMI, and anticholinergic risk score; feature set 2) and the replacement of the 3-category MPI in feature sets 1 and 2 with the eight separate MPI domains (feature sets 3 and 4, respectively), and to assess the prediction accuracy of the ML algorithms using the same feature sets.

**Methods:**

MPI and clinical features were collected from patients aged 65 years and above who were admitted to either the general medical or acute care of the elderly wards of a South Australian hospital between September 2015 and February 2017. The diagnostic accuracy of LR-MLE was assessed together with nine ML algorithms: decision trees, random forests, extreme gradient boosting (XGBoost), support-vector machines, naïve Bayes, K-nearest neighbors, ridge regression, logistic regression without regularization, and neural networks. A 70:30 training set:test set split of the data and a grid search of hyper-parameters with 10-fold cross-validation—was used during model training. The area under the curve was used as the primary measure of accuracy.

**Results:**

A total of 737 patients (female: 370/737, 50.2%; male: 367/737, 49.8%) with a median age of 80 (IQR 72-86) years had complete MPI data recorded on admission and had completed the 12-month follow-up. The area under the receiver operating curve for LR-MLE was 0.632, 0.688, 0.738, and 0.757 for feature sets 1 to 4, respectively. The best overall accuracy for the nine ML algorithms was obtained using the XGBoost algorithm (0.635, 0.706, 0.756, and 0.757 for feature sets 1 to 4, respectively).

**Conclusions:**

The use of MPI domains with LR-MLE considerably improved the prediction accuracy compared with that obtained using the traditional 3-category MPI. The XGBoost ML algorithm slightly improved accuracy compared with LR-MLE, and adding clinical data improved accuracy. These results build on previous work on the MPI and suggest that implementing risk scores based on MPI domains and clinical data by using ML prediction models can support clinical decision-making with respect to risk stratification for the follow-up care of older hospitalized patients.

## Introduction

### Background

Previous studies have highlighted the importance of using functional measures to predict mortality among older hospitalized patients, a complex population characterized by different degrees of frailty, comorbidity burden, and polypharmacy [1,2]. In particular, the Multidimensional Prognostic Index (MPI), an objective and quantifiable comprehensive geriatric assessment (CGA)–based tool developed from 8 separate CGA domains, is an example of a cumulative deficit model of frailty [3]. The MPI is strongly associated with mortality [2], length of hospital stay [4], and hospital readmission [3] in subpopulations suffering from acute and chronic conditions, including general and geriatric hospital patients. The MPI patient score is created by aggregating the total scores from the 8 separate CGA tools and normalizing the resulting total score to provide a value in the range from 0 to 1. The latter is then categorized into three categories of risk: low (0.0-0.33), moderate (0.34-0.66), and severe (0.67-1.0), allowing clinicians to better tailor their care management.

Prediction accuracy for 12-month mortality (12MM) using the area under the receiver operating curve (AUC) of 0.751 was achieved with the three MPI categories when validated in an older Italian population [2]. However, despite it being a significant predictor of 12MM in a similar older hospital population within Australia [5], its diagnostic accuracy was not confirmed (AUC=0.64, with age and gender adjustment). The relatively poor performance for prediction compared with the prediction accuracy with the Italian cohort might be partly explained by the homogenization of scores from the separate MPI domains into a single aggregate-weighted scoring system. Although this simplifies risk classification, the use of an aggregate-weighted scoring system has been shown in general to remove important domain-specific information, resulting in poorer risk prediction [6]. Conversely, the use of individual MPI domains in place of an aggregate score has the potential to cause overfitting of the prediction model (ie, reduced bias), which results in lower accuracy when used on independent data sets (ie, increased variance) [7].

Machine learning (ML) is a branch of artificial intelligence in which various algorithms are used to make predictions [8]. The algorithms differ from standard statistical modeling approaches such as those using least squares or maximum likelihood, which focus on linear relationships and have no additional aspects to their respective error functions (such as the use of regularization) that help reduce the likelihood of model overfitting. The strengths of ML algorithms include their ability to handle feature selection in the presence of collinearity, and the ability to deal with a larger number of features including complex nonlinear patterns and interactions [9]. Furthermore, the validation of ML-based approaches is generally more rigorous than that of the standard statistical approaches, with special care taken to consider the aforementioned trade-off between bias and variance when developing the prediction model during the training process [10]. Finally, model development is more data driven; it does not rely solely on content knowledge, thereby increasing the opportunity to identify previously unconsidered features for enhanced prediction [8].

### Objective

Given the relatively poor performance of the aggregate MPI score in predicting 12MM in an Australian cohort, we seek to improve the prediction accuracy in several ways. First, we use the separate components of the MPI as input features for a traditional logistic regression with maximum likelihood estimation (LR-MLE). Second, we assess nine different binary-classification ML algorithms that might perform better than LR-MLE. Third, we add 10 routinely collected clinical measures to the MPI domain-based feature data set.

## Methods

### Overview

Details of the data collection methods, including a description of the study design, study cohort, and collected data, have been previously published [5]. Briefly, the cohort consisted of patients aged 65 years and above admitted to the Flinders Medical Centre Acute Medical Unit and then transferred to either the general medical or acute care of the elderly wards between September 14, 2015, and February 17, 2017. Flinders Medical Center is a 593-bed metropolitan teaching and trauma hospital within the Southern Adelaide Local Health Network, which has a catchment area of approximately 350,000 people. Acute care of the elderly wards provides a comprehensive individualized approach for assessing older frail medical inpatients using a multidisciplinary team. The study was conducted in accordance with the Declaration of Helsinki and the guidelines for Good Clinical Practice. Approval for the study was obtained from the local ethics committee (reference number: 170.15).

### Feature Sets

The 63-item MPI is a prognostic tool based on averaging the standardized scores obtained from the eight core domains of the CGA, which were obtained in all study participants within the first 3 days of hospital admission. We trained the prediction models using four different feature sets. The first feature set contained age, gender, and the 3-category MPI as three separate dummy variables (n=5 features in total). The second feature set contained the five features from the first feature set as well as 10 additional clinical features: BMI, anticholinergic risk score (ARS) [11], serum sodium, hemoglobin, serum albumin, creatinine, urea, urea-to-creatinine ratio, estimated glomerular filtration rate (eGFR), and C-reactive protein (CRP; n=15 features in total). The third and fourth feature sets included the eight separate MPI domains used in the calculation of the overall MPI score, in place of the 3-category MPI used in the first and second feature sets, resulting in n=10 and n=20 features, respectively. The eight MPI domains consist of cohabitation status (living alone, with family or friends, or in an institute), the total number of prescribed medications (taken at admission), functional status evaluated with activities of daily living (ADL) and instrumental ADL (IADL) scales; cognitive status evaluated by the Short Portable Mental Status Questionnaire; evaluation of pressure sores using the Exton Smith Scale (ESS); comorbidities assessed using the Cumulative Illness Rating Scale (CIRS); and nutritional status evaluated by the Mini Nutritional Assessment (MNA). The additional biochemical features are known to be associated with adverse outcomes in older patient populations [12-18]. The target variable for the prediction models was all-cause mortality within 12 months (12MM), defined as death from any cause and obtained using the Australian national death registry.

### ML Algorithms

We implemented a systematic ML-based framework to construct the 12MM prediction models. The steps included data preprocessing, splitting of the data into training and validation data sets, model development using the training data set for each algorithm, and final assessment of the accuracy of each algorithm using the validation data set*.* The data preprocessing step included imputation of missing values and the scaling of continuous features, which included all features except for gender, the 3-category MPI, and cohabitation status. Any continuous features with missing values were imputed using the mean value of that feature before scaling the features and data splitting. Continuous features were scaled to have a zero mean and unit variance. Following preprocessing, the data were split into a training data set for the development of the prediction models and a test data set for accuracy validation. Data were split randomly into two sets in the ratio 70:30 with the 69.9% (515/737) sample defined as the training set and used for development of the prediction models, and the 30.1% (222/737) sample defined as the test (ie, validation) set and used to validate the accuracy of the algorithm. Once the training set was defined, an optimal model was developed for nine different ML algorithms: decision trees (DTs), random forests (RFs), eXtreme Gradient Boosting (XGBoost), support vector machines (SVM), naïve Bayes, K-nearest neighbors (KNN), ridge regression (logistic regression with *L2* regularization), logistic regression without regularization, and neural networks (NNs). A description of each algorithm is provided in Multimedia Appendix 1. Multimedia Appendix 2 provides the data set for the study and Multimedia Appendices 3-7 contain the Python code for all of the analysis performed for this study. All features were used without creating interactions or higher-order terms. However, as a sensitivity analysis, we also assessed each algorithm using all linear and second-order polynomial terms, that is, after squaring each feature and including all 2-way interactions.

For each algorithm, a grid search of hyper-parameters was performed to find the optimal set of hyper-parameters for training data accuracy. Each grid search was performed using 10-fold cross-validation, in which the training data set was split into 10 equally sized discrete folds. A model was then created using 90% (9/10 folds) of the data, and its accuracy was assessed using the remaining fold of data. The process was repeated 10 times, with each fold held out for one of the 10 training steps and used to assess the model accuracy for the training data. The AUC was used as the accuracy metric during the grid search. Once the optimal set of hyper-parameters was defined for each of the algorithms based on the training data, the performance of the optimal model for each ML algorithm was assessed on the test data set using the AUC as the primary accuracy metric. Although numerous accuracy measures are available for ML algorithms, the AUC is the most used in clinical settings and allows comparison with other studies, and we therefore used this as our primary accuracy measure. However, for completeness, we also report accuracy, precision, recall, and F1 score, given the imbalance in the number of positive and negative outcomes (dead and alive patients) that can, by itself, lead to higher AUC values [19].

### Logistic Regression With Maximum Likelihood Estimation

For each feature set, the estimated logit coefficients obtained for the LR-MLE using the training data set were used to predict and assess the accuracy of the model on the test data set. The odds ratios (ORs), 95% CIs, and *P* values for the training data set with feature set 4 are reported.

### Statistical Analysis

All analyses were performed using Python version 3.8.3. The normal distribution of the features was assessed using quantile-quantile plots and histograms, and descriptive statistics, including the mean, median, or frequency, were used for each feature as appropriate. Between-group comparisons for those alive and deceased at 12 months were performed using two-tailed independent *t* tests, Mann-Whitney tests, or chi-square tests. Each ML algorithm was implemented using Python’s scikit-learn library [20], except for the XGBoost algorithm, which has its own Python package [21]. Relative importance feature plots and calibration plots were produced for the best algorithm, and violin plots were used to describe the distribution of the most important features. The calibration of the best-performing algorithm was shown by plotting the observed versus predicted deciles of risk. LR-MLE was performed using the logit function of Python’s *statsmodels* module, and the LR-MLE models included all features within each feature set. Descriptive statistics were analyzed using the SciPy library (version 1.4.1) *stats* module, and plots were drawn using the *matplotlib* and *seaborn* libraries.

### Data Sharing Statement

All data generated or analyzed during this study are included in Multimedia Appendix 2. The Python code used to analyze the data is also available in the Multimedia Appendices 3-7.

## Results

### Overview

The cohort included a total of 737 patients that were each assessed for MPI and followed up for 12 months. There were no missing values for age, sex, or each of the MPI domains. Among the additional five variables used in feature set 4, there were a total of 66 missing values, including sodium (n=1) albumin (n=3) hemoglobin (n=2), urea (n=1) creatinine (n=1), urea-to-creatinine ratio (n=1), eGFR (n=1), and CRP (n=56). Table 1 describes the characteristics of the patients according to their vital status at 12 months after mean imputation for missing values. There were significant differences between the two groups for all 20 features except for sex, the total number of medications used, the Short Portable Mental Status Questionnaire score, eGFR, creatinine, and the ARS.

**Table 1:** Patient characteristics according to vital status at 12 months after hospital discharge.

Characteristics			Alive (n=536)		Deceased (n=201)		*P* value^a^	
Age (years), median (IQR)			79 (72-85)		82 (74-88)		.002	
**Gender, n (%)**								.14
	Female	278 (51.9)		92 (45.6)				
	Male	257 (47.9)		110 (54.4)				
**MPI^b^ category, n (%)**								<.001
	Mild	211 (39.4)		39 (19.3)				
	Moderate	290 (54.2)		136 (67.3)				
	Severe	34 (6.4)		27 (13.4)				
**MPI domains**								
	ADL^c^, median (IQR)	6 (5-6)		5 (4-6)		<.001		
	IADL^d^, median (IQR)	6 (4-8)		4 (3-6)		<.001		
	SPMSQ^e^, median (IQR)	1 (0-2)		1 (0-3)		.05		
	ESS^f^, median (IQR)	18 (17-19)		17 (15-18)		<.001		
	CIRS^g^, mean (SD)	2.4 (0.4)		2.6 (0.4)		<.001		
	MNA^h^, mean (SD)	20.9 (3.9)		18.0 (4.6)		<.001		
Total number of medications, mean (SD)			10.0 (4.4)		10.3 (4.5)		.55	
**Cohabitation status, n (%)**								.009
	Living alone	199 (37.2)		70 (34.7)				
	Family or friends	300 (56.1)		104 (51.5)				
	Institute	36 (6.7)		28 (13.9)				
BMI (kg/m^2^), median (IQR)			26.9 (23.8-31.8)		25.2 (22.0-29.1)		<.001	
Sodium (mmol/L), median (IQR)			138 (135-140)		138 (135-140)		.006	
Albumin (g/L), mean (SD)			32.5 (5.6)		30.4 (5.7)		<.001	
Hemoglobin (g/L), mean (SD)			118.7 (18.2)		112.0 (18.3)		<.001	
eGFR^i^ (mL/min/1.73m^2^), mean (SD)			55.3 (24.2)		52.2 (26.6)		.14	
CRP^j^ (mg/L), median (IQR)			29.0 (6.0-81.0)		33.0 (14.2-84.0)		.048	
Creatinine (mmol/L), median (IQR)			95 (74-134)		103 (72-151)		.09	
Urea (mmol/L), median (IQR)			7.40 (5.3-11.6)		8.90 (5.7-15.0)		<.001	
Urea-to-creatinine ratio, median (IQR)			0.08 (0.06-0.10)		0.09 (0.06-0.10)		.001	
ARS^k^, median (IQR)			0 (0-2)		0 (0-2)		.41	

^a^Using two-tailed independent *t* test, Mann-Whitney U test, or chi-square test, as appropriate.

^b^MPI: Multidimensional Prognostic Index.

^c^ADL: activities of daily living.

^d^IADL: instrumental activities of daily living.

^e^SPMSQ: Short Portable Mental Status Questionnaire.

^f^ESS: Exton Smith Scale.

^g^CIRS: Cumulative Illness Rating Scale.

^h^MNA: Mini Nutritional Assessment.

^i^eGFR: estimated glomerular filtration rate.

^j^CRP: C-reactive protein.

^k^ARS: anticholinergic risk score.

### Correlation Matrix Heatmap

**Figure 1:**
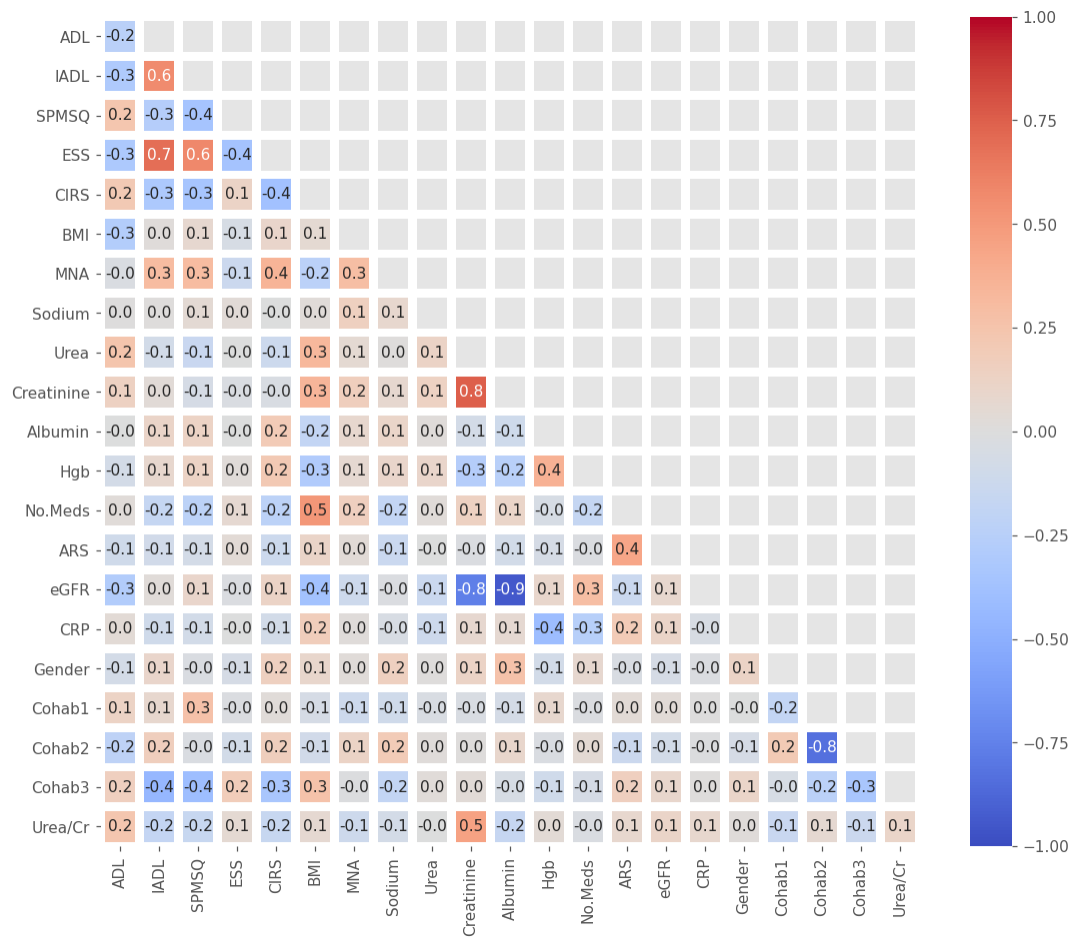
Spearman ρ correlation matrix heatmap for feature set 4. ADL: activities of daily living; ARS: anticholinergic risk score; CIRS: Cumulative Illness Rating Scale; Cohab1: living alone; Cohab2: living with family or friends; Cohab3: living in an institute; CRP: C-reactive protein; eGFR: estimated glomerular filtration rate; ESS: Exton Smith Scale; Hgb: serum hemoglobin; IADL: instrumental activities of daily living; MNA: Mini Nutritional Assessment; No.Meds: number of medications; SPMSQ: Short Portable Mental Status Questionnaire; Urea/Cr: urea-to-creatinine ratio.

Figure 1 shows the Spearman ρ correlation matrix heatmap for features in feature set 4. Moderate to strong positive correlations were observed between creatinine and urea (ρ=0.75), ADL and ESS (ρ=0.69), IADL and ESS (ρ=0.58), ADL and IADL (ρ=0.57), and between CIRS and number of medications (ρ=0.51). There were also strong to moderate negative correlations between cohabitation status 1 and 2, that is, living alone and living with family or friends (ρ=−0.84), eGFR and creatinine (ρ=−0.94), eGFR and urea (ρ=−0.77), and CRP and albumin (ρ=−0.41). The absolute strengths of all other correlations were │ρ│≤0.40. The lack of many highly correlated features suggested that the use of data reduction techniques such as principal component analysis before modeling was unnecessary.

### ML Algorithms

#### Test Data Accuracy

**Figure 2:**
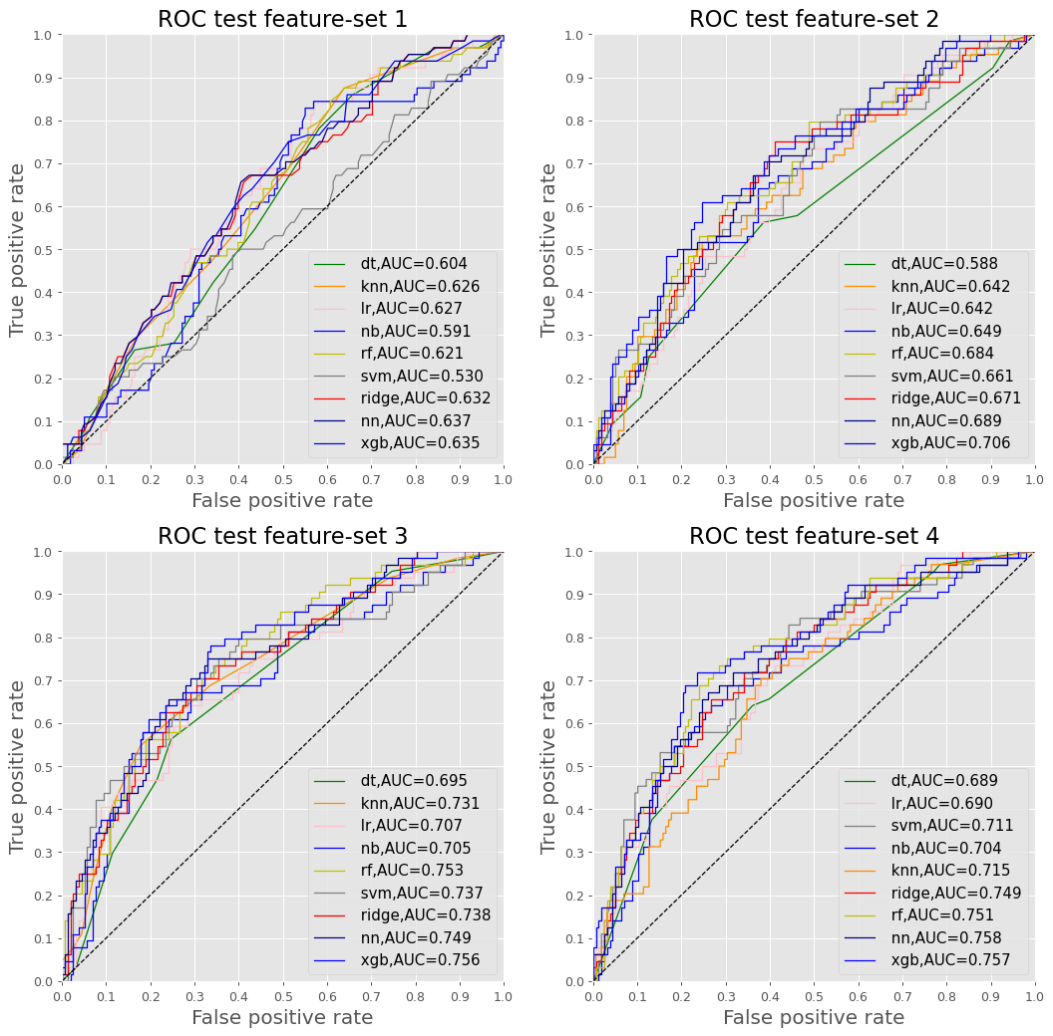
Test accuracy of the 9 machine learning algorithms using feature sets 1 to 4. AUC: area under the receiver operating curve; dt: decision tree; knn: K-nearest neighbors; lr: logistic regression without penalization; nb: naive bayes; nn: neural network; rf: random forest; ridge: ridge regression; ROC: receiver operating curve; svm: support vector machine; xgb: eXtreme gradient boosting.

Table 2 and Figure 2 describe the test accuracy results for the four feature sets. The AUC for LR-MLE for feature sets 3 and 4 (0.738 and 0.757, respectively) that contained the eight MPI domains were considerably higher than those for feature sets 1 and 2 (0.632 and 0.688, respectively) that contained the three MPI categories. The AUC for LR-MLE was lower than those for at least one of the ML algorithms for each feature set 1 to 3 and was very similar to the best ML algorithms for feature set 4 (0.757 for LR-MLE vs 0.757 for XGBoost and 0.758 for NN). Overall, the best-performing ML algorithm was XGBoost, with an AUC ranging from 0.635 to 0.757 for feature sets 1 and 4, respectively, and a mean AUC of 0.714 for all four feature sets. The AUC for LR-MLE and all nine ML algorithms was improved with the addition of the clinical data (feature set 2 vs feature set 1 and feature set 4 vs feature set 3). The AUC was also improved for some, but not all, of the ML algorithms with the addition of clinical data. Multimedia Appendix 8 provides the results of accuracy, precision, recall, and F1 score for the LR-MLR model and the nine ML algorithms. As with the AUC, values for accuracy and precision were comparable across the various models with a range of 0.707 (KNN) to 0.752 (NN) for accuracy and a range of 0.474 (KNN) to 0.654 (SVM) for precision. However, there was a wider variability for recall and F1 scores with recall ranging from 0.031 (DT) to 0.531 (naïve Bayes) and the F1-score ranging from 0.059 (DT) to 0.751 (SVM).

**Table 2:** Diagnostic accuracy for logistic regression with maximum likelihood estimation and the 9 machine learning algorithms using feature sets 1 to 4 with the test data set.

Model		AUC^a^				
		Feature set 1^b^	Feature set 2^c,d^	Feature set 3^e^	Feature set 4^f,d^	Value, mean (SD)
LR-MLE^g^		0.632	0.688	0.738	0.757	0.704 (0.06)
**Machine learning algorithms**						
	XGB^h^	0.635	0.706	0.756	0.757	0.714 (0.06)
	Neural network	0.637	0.689	0.749	0.758	0.708 (0.06)
	Random forest	0.621	0.684	0.753	0.751	0.702 (0.06)
	Ridge^i^	0.632	0.671	0.738	0.749	0.698 (0.06)
	KNN^j^	0.626	0.642	0.731	0.715	0.679 (0.06)
	Nonpenalized logistic regression	0.627	0.642	0.707	0.690	0.667 (0.05)
	Naïve Bayes	0.591	0.649	0.705	0.704	0.663 (0.04)
	SVM^k^	0.530	0.661	0.737	0.711	0.656 (0.09)
	Decision tree	0.604	0.588	0.695	0.686	0.643 (0.06)

^a^AUC: area under the receiver operating curve.

^b^Multidimensional Prognostic Index categories, age, gender (n=5 features).

^c^Multidimensional Prognostic Index categories, age, gender, BMI, anticholinergic risk score, laboratory data (n=15 features).

^d^Lab data=serum albumin, sodium, serum hemoglobin, C-reactive protein, creatinine, urea, urea-to-creatinine ratio, and estimated glomerular filtration rate.

^e^Multidimensional Prognostic Index domains, age, gender (n=10 features).

^f^Multidimensional Prognostic Index domains, age, gender, BMI, anticholinergic risk score, laboratory data (n=20 features).

^g^LR-MLE: logistic regression with maximum likelihood estimation.

^h^XGB: extreme gradient boosting.

^i^Ridge: ridge regression.

^j^KNN: K-nearest neighbors.

^k^SVM: support vector machine.

#### Training Data Accuracy

Multimedia Appendices 9 and 10 show the AUC values for LR-MLE and each ML algorithm for feature sets 1 to 4 using the training data set. The accuracy for LR-MLE was slightly higher for each feature set with the training data than for the test data values shown in Table 2. In comparison, the accuracy for each ML algorithm was considerably higher than that obtained using the test data, especially for the RF, XGB, and SVM, which obtained values of 0.956, 0.877, and 0.855, respectively, using feature set 4 and the training data.

#### Calibration

Multimedia Appendix 11 shows the distribution of predicted risk scores, the calibration plot, precision-recall curve, and receiver operating characteristic curve for the XGBoost algorithm using feature set 4. The calibration plot showed that overall, the predicted risks of mortality were in line with each observed risk decile. An AUC of 0.757 indicates fair to good accuracy in terms of overall sensitivity and specificity. The precision-recall curve indicates that the precision (ie, sensitivity or the ability to identify the patients that died) gradually decreased as the threshold for positivity decreased, and the recall, that is, the value of a positive classification, increased.

#### Feature Importance and Distributions

**Figure 3:**
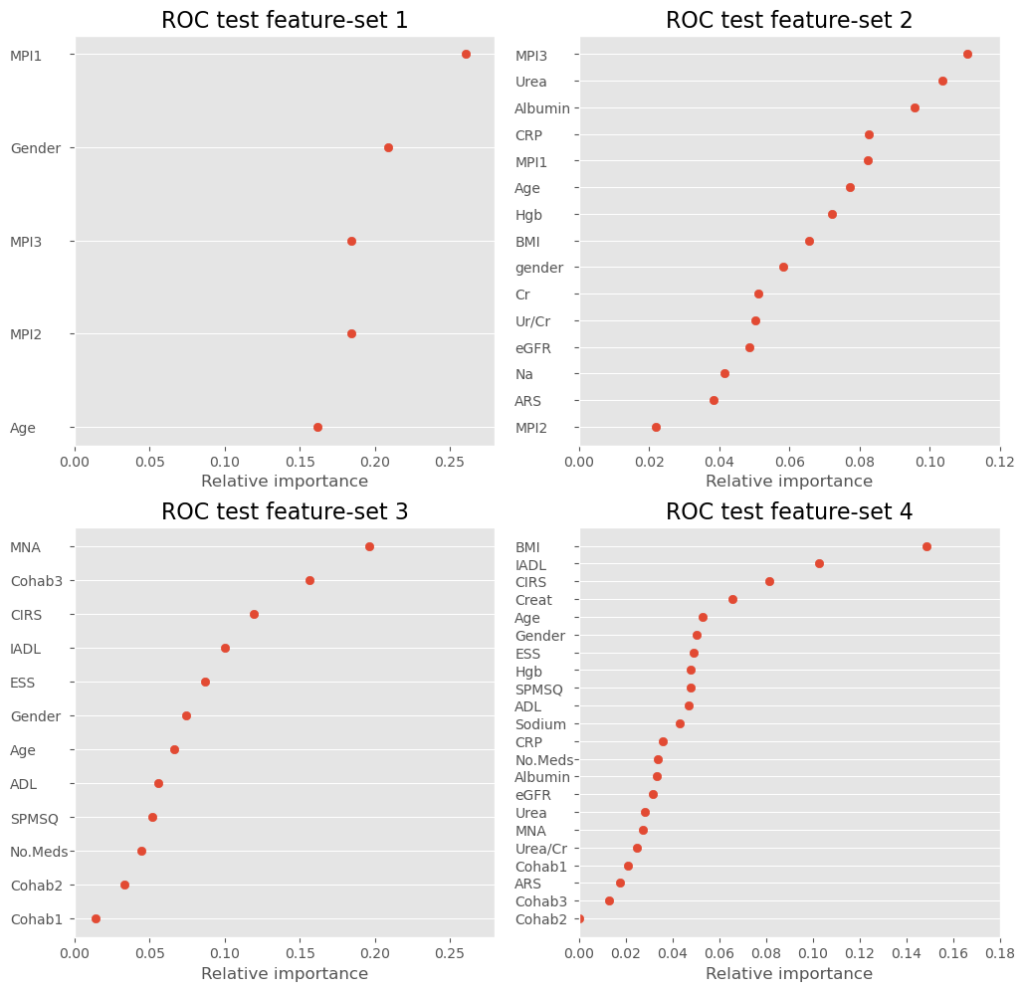
Feature importance plot for the eXtreme gradient boosting algorithm using test data with feature sets 1 to 4. ADL: activities of daily living; ARS: anticholinergic risk score; CIRS: Cumulative Illness Rating Scale; Cohab1: living alone; Cohab2: living with family or friends; Cohab3: living in an institute; Creat: creatinine; CRP: C-reactive protein; eGFR: estimated glomerular filtration rate; ESS: Exton Smith Scale; Hgb: serum hemoglobin; IADL: instrumental activities of daily living; MNA: Mini Nutritional Assessment; MPI: Multidimensional Prognostic Index; MNA: Mini Nutritional Assessment; ROC: receiver operating curve; SPMSQ: Short Portable Mental Status Questionnaire; Ur/Cr: urea-to-creatinine ratio.

**Figure 4:**
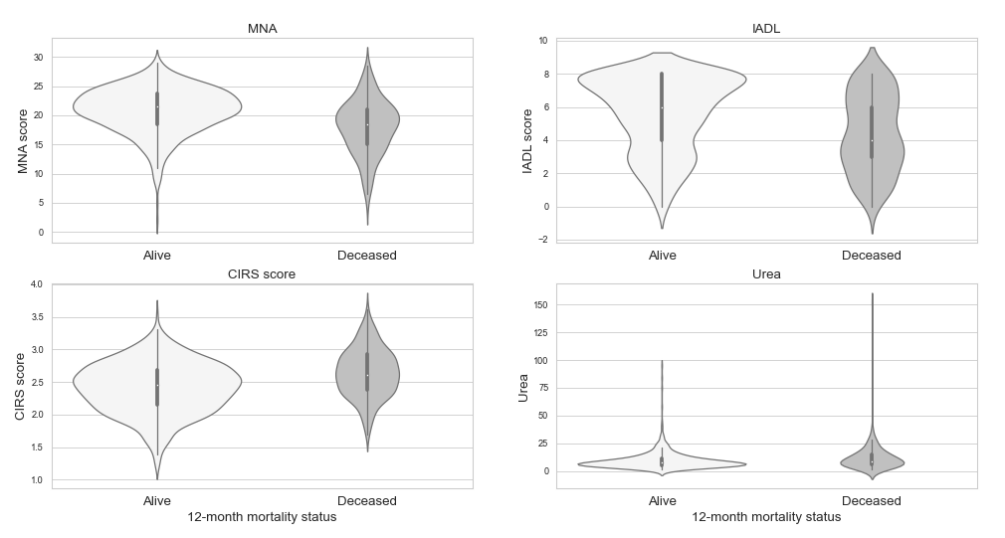
Violin plots showing distributions for the top 4 features for eXtreme gradient boosting in the second test feature set by patient vial status at 12 months after hospital discharge. CIRS: Cumulative Illness Rating Scale; IADL: instrumental activities of daily living; MNA: Mini Nutritional Assessment.

Figure 3 shows the feature importance plots for the XGBoost algorithm for the test data set using feature sets 1 to 4. In feature set 4, the MNA, IADL, and CIRS domains had the highest feature importance, indicating that they had the largest relative importance among the included features. Living alone (Cohab1) and urea were also highly ranked. The violin plots in Figure 4 show the distribution of MNA, IADL, and CIRS domains and urea—the four continuous features that accounted for the highest relative importance for the XGBoost algorithm in test feature set 4. The shape of the distributions was markedly different among patients who remained alive and those who died for the MNA score and the IADL score, which together accounted for 26.4% of the relative importance. The distributions were more similar for the CIRS score and urea, which accounted for 7.5% and 6.1% of the relative importance, respectively.

#### LR-MLE Accuracy

Table 3 shows the standardized odds ratios, 95% CIs, and *P* values for the LR-MLE model using test feature set 4. The variables that were statistically significant also had the largest standardized effect sizes: the MNA (OR 0.57, 95% CI 0.44-0.74; *P*<.001), CIRS domain (OR 1.81, 95% CI 1.32-2.49; *P*<.001), and the number of medications prescribed (OR 0.69, 95% CI 0.52-0.93; *P*=.02).

**Table 3:** Odds ratios (95% CIs) for the logistic regression with maximum likelihood estimation model using the test data with feature set 4^a^.

Feature		Odds ratio (95% CI)	*P* value
Age		1.20 (0.90-1.61)	.21
ADL^b^		0.99 (0.71-1.39)	.96
IADL^c^		0.88 (0.63-1.22)	.44
SPMSQ^d^		0.99 (0.78-1.25)	.91
ESS^e^		0.89 (0.62-1.27)	.51
CIRS^f^		1.81 (1.32-2.49)	<.001
BMI		0.82 (0.63-1.07)	.14
MNA^g^		0.57 (0.44-0.74)	<.001
Sodium		0.96 (0.77-1.20)	.74
Urea		1.77 (0.89-3.51)	.10
Creatinine		0.85 (0.53-1.36)	.50
Albumin		0.83 (0.64-1.07)	.15
Hemoglobin		0.88 (0.68-1.14)	.35
Number of medications		0.69 (0.52-0.93)	.02
ARS^h^		1.06 (0.82-1.36)	.66
eGFR^i^		1.15 (0.74-1.80)	.53
CRP^j^		0.94 (0.73-1.20)	.61
**Cohabitation**			
	Alone	1.00^k^	N/A^l^
	Family or friends	0.96 (0.73-1.25)	.75
	Institute	1.22 (0.95-1.57)	.12
**Gender**			
	Female	1.00^k^	N/A
	Male	0.69 (0.40-1.18)	.18
Urea-to-creatinine		1.13 (0.67-1.91)	.64

^a^All continuous variables were scaled before analysis to have a mean of zero and an SD of 1. Gender and cohabitation status were dummy coded for each category.

^b^ADL: activities of daily living.

^c^IADL: instrumental activities of daily living.

^d^SPMSQ: Short Portable Mental Status Questionnaire.

^e^ESS: Exton Smith Scale.

^f^CIRS: Cumulative Illness Rating Scale.

^g^MNA: Mini Nutritional Assessment.

^h^ARS: anticholinergic risk score.

^i^eGFR: estimated glomerular filtration rate.

^j^CRP: C-reactive protein.

^k^This is the reference group. Therefore, there is no CI.

^l^N/A: not applicable.

### Sensitivity Analysis

In the sensitivity analysis that included all features as both first- and second-order terms, all results for the AUC for the nine ML algorithms were very similar to those obtained using only first order terms (data not shown).

## Discussion

### Principal Findings

In this study, we applied a range of ML binary-classification algorithms to data from 737 older inpatients in an Australian teaching hospital to develop and test a 12MM prediction model that can potentially be used in clinical settings to assist with risk management. The test data accuracy using the eight MPI domains and age and gender reached an AUC of 0.738 using LR-MLE, considerably higher than that obtained using the MPI categories plus age and gender (AUC=0.632). The addition of 10 clinical features improved the prediction accuracy further to AUC=0.757, which matched the accuracy obtained for the best overall ML-based algorithm (XGBoost), which outperformed most of the other algorithms except for the largest feature set in which the NN algorithm had similar accuracy (AUC=0.758).

The major strengths of our study include the use of both LR-MLE and a wide range of commonly used ML algorithms to compare the prediction accuracy for aggregate versus domain-based data. In the development of the ML algorithms, we also used a systematic ML framework, with a grid search of the hyperparameter space and 10-fold cross-validation for each algorithm. The much-improved accuracy warrants the calculation of individual patient risk scores, which, with appropriately developed technology platforms linked to the MPI domain and clinical information, can be used to better stratify patient risk and provide appropriate posthospital discharge surveillance and care [22,23].

### Comparison With Prior Work

The prediction accuracy using the MPI categories and the test data set (AUC=0.632) was very similar to the poor accuracy obtained previously with follow-up on only 697 of the same patients (AUC=0.62), in which all records were used for assessing prediction accuracy rather than using separate training and testing data sets [5]. However, the higher accuracy obtained using the eight individual component features of the MPI combined with clinical data led to an accuracy similar to that originally reported for the three-category MPI within the original Italian MPI cohort [2]. In addition, the improvement in prediction accuracy with ML algorithms for some but not all feature sets provides general support for the use of ML in addition to LR-MLE when developing risk scores, at least for moderately sized data sets and feature numbers. The accuracy values obtained for precision, recall, and F1 score, which are less subject to variation than the AUC in imbalanced data sets, were of the same order for LR-MLE and the XGBoost algorithm and within the upper range of values obtained for these metrics.

The significantly higher accuracy obtained by using the separate domains of the MPI compared with using the 3-category aggregate MPI supports other studies in which the use of component domain data outperformed an aggregate score. In a meta-analysis of 6 studies comparing individual domain feature input to aggregate weighted scores for mortality and intensive care unit transfer, prediction using ML algorithms or multivariate regression with separate features considerably enhanced prediction compared with that obtained using the aggregate scoring systems (AUC=0.80 vs AUC=0.73) [6]. These findings suggest that caution should be used when employing aggregate risk scoring systems and the need to consider the underlying individual components. In addition, in our study, certain MPI domains, including the MNA, IADL, ESS, and CIRS, had either strong feature importance using the XGBoost algorithm or were strongly associated with 12MM using LR-MLE. Therefore, it may also be possible to obtain predictive accuracy similar to or better than that of the 3-category MPI feature sets in this study, by using data collected for only a subset of the eight MPI domains and by using the individual items for these specific domains. However, such an approach requires validation using data collected from additional retrospective and/or future prospective cohorts.

Unlike many ML algorithms that attempt to reduce the potential for overfitting and increased variance, the traditional LR-MLE approach to prediction modeling is not implicitly designed to deal with bias, multicollinearity, nonlinearity, or feature interactions. Thus, although the addition of features and model complexity generally improves a model’s performance during training, a new and larger model does not guarantee similar improvements in model fit in the validation phase. Indeed, without some form of additional penalty term in the model’s loss function to ensure that the training model is not being overfit, a decrease in testing accuracy is not uncommon [24]. When an additional *L2* penalty term was applied to the Logistic Regression classifier, the resulting ridge regression ML classification algorithm provided a slight increase in prediction accuracy for all four test feature sets (mean AUC of 0.698 for ridge regression vs AUC of 0.667 for nonregularized logistic regression). This reduction in strength (ie, regularization) of the estimated parameters for the ridge regression and the other ML algorithms during the training phase of model development may partly explain the higher test accuracy for LR-MLE in which there is no equivalent model training or an additional penalty term to help ensure that the LR-MLE coefficients are not inflated and that residual error is not overly reduced. Similarly, although many of the ML algorithms had better training performance than the LR-MLE, this did not translate into better test performance, suggesting that these ML algorithms often overfit the data during training, a problem that could potentially be solved with greater tuning of the algorithm’s hyper-parameters.

A common criticism of ML approaches compared with standard statistical approaches is that they rely on blackbox algorithms in which the source of the improved performance is not readily transparent [25,26]. One method to overcome this lack of transparency is through the calculation of feature importance, which shows the relative importance of each feature in terms of reducing prediction error [26]. By using this method, we demonstrated the very high relative importance of the MNA and IADL domains of the MPI regarding risk for 12MM. Thus, although the ML algorithms do not generally provide a single specific effect estimate and CI for each variable or feature, our findings support other studies in which the MNA [27] and the IADL [28] have been shown to be independently and strongly associated with medium-term mortality in similar cohorts. These two features were also the strongest independent features in the LR-MLE model using feature set 4, emphasizing their importance in 12MM prediction.

The independent predictive ability of functional, cognitive, and psychological measures in predicting mortality in older hospitalized patients has been previously identified, with the IADL, mini mental state examination, and geriatric depression scale all shown to be independent predictors of mortality after risk adjustment for clinical characteristics [1]. However, the prediction accuracy in that study was modest (AUC=0.690), suggesting that complementary clinical data may have further improved prediction. The improvement in performance with the addition of blood biochemistry data, BMI, and ARS in our study highlights the independent predictive value of clinical data. Many of these features are known markers of malnutrition or frailty [29,30] and are associated with poorer outcomes in older patients [12,31,32]. Their independence with the MPI domains is demonstrated by weak univariate correlations, indicating their potential to add predictive power to that obtained from the domains. As each biochemical marker is routinely measured in hospital laboratories, it is highly feasible to incorporate them into hospital-based prediction algorithms.

Our results for the ML algorithms, based on a rigorous train-test validation approach with a grid search of hyper-parameters for each algorithm during training, demonstrate the potential of such methods in predicting mortality in clinical settings. In particular, the XGBoost algorithm, which is known as a superior and fast prediction ML algorithm, had the highest overall level of accuracy for the feature sets using the validation data set, and one of the highest levels of accuracy using the training data set, suggesting that the prediction accuracy observed for our cohort is likely to be repeatable in other cohorts with a similar patient profile. A distinguishing feature of the XGBoost algorithm is its approach of building a *strong learner* from an ensemble of *weak learners* (ie, separate DTs) by building successive trees, in which additional weight is placed on harder to predict subsets of the training data [33]. XGBoost uses a second-order Taylor series expansion to approximate the value of the loss function and incorporates regularization to avoid overfitting [21]. These features together ensure a fast solution as well as an unbiased estimate of prediction accuracy, both of which are important in building an automated risk prediction model at a system level within a clinical setting.

### Limitations

Our study had several limitations. In using only routine geriatric assessment tools and basic biochemical, medication, and demographic data, it is likely that we were limited in our ability to obtain higher levels of predictive accuracy. The development of better prediction models should be possible using a richer set of features, such as additional information on demographics, comorbidities, laboratory test measurements, and medication type. Specifically, the Charlson Comorbidity Index, which determines the risk of medium-term mortality due to a specific set of 17 different comorbidities, is generally readily calculable using the secondary diagnosis codes captured in most administrative databases [34]. Similarly, routine hospital blood tests capture biomarker data associated with 12MM [35], and most hospitals capture medication data that can be used to assess the predictive capacity of medication type, dose, and polypharmacy. Other potentially predictive features include the number of hospitalizations and GP visits in the previous 12 months [36]. Our study data were also limited by its relatively small size, given that the test data set included only 222 patients and 64 deaths. ML methods have been found to generally require larger data sets, and particularly a higher number of events before stable measures of prediction performance are obtained in comparison with standard statistical models [37]. Despite this limitation, we still achieved moderate to good validation accuracy using data sets with a relatively limited number of events. In addition, although we were careful to use cross-validation throughout the model training process and to validate each model in a held-out test data set, we cannot be sure that our prediction accuracy will be the same in different cohorts of the same patient population or in different older populations such as persons living in aged care facilities. Further validation of the models in additional retrospective cohorts as well as prospective cohorts is required to ensure generalizability.

### Conclusions

An MPI domain-based approach, together with clinical and demographic data, improved the prediction of mortality compared with a logistic regression model that used the aggregate MPI score. The ML algorithms in this study generally provided improved prediction accuracy compared with LR-MLE. These results build on previous work for the MPI and suggest that implementing risk scores based on MPI domains and clinical data with ML prediction models can be used to support clinical decision-making with respect to the medium-term follow-up care of older hospitalized patients.

## Appendix

Multimedia Appendix 1 The machine learning algorithms.

Multimedia Appendix 2 The dataset Multidimensional Prognostic Index feature set 4.

Multimedia Appendix 3 Python code for correlation matrix.

Multimedia Appendix 4 Python code for XGBoost feature importance.

Multimedia Appendix 5 Python code for receiver operating curve.

Multimedia Appendix 6 Python code for violin plots.

Multimedia Appendix 7 Python code for machine learning algorithms.

Multimedia Appendix 8 Diagnostic accuracy with the training data set.

Multimedia Appendix 9 Table S2. Accuracy, precision recall, F1 score.

Multimedia Appendix 10 Training accuracy of the 9 machine learning algorithms using feature sets 1 to 4.

Multimedia Appendix 11 Plots showing predicted risk distribution, calibration curve, precision-recall curve, and the area under the curve for the eXtreme gradient boosting algorithm using test data and feature set 4.

## Abbreviations

12MM: 12-month mortality
ADL: activities of daily living
ARS: anticholinergic risk score
AUC: area under the receiver operating curve
CGA: comprehensive geriatric assessment
CIRS: Cumulative Illness Rating Scale
CRP: C-reactive protein
DT: decision tree
eGFR: estimated glomerular filtration rate
ESS: Exton Smith Scale
IADL: instrumental activities of daily living
KNN: K-nearest neighbors
LR-MLE: logistic regression with maximum likelihood estimation
ML: machine learning
MNA: Mini Nutritional Assessment
MPI: Multidimensional Prognostic Index
NN: neural network
OR: odds ratio
RF: random forest
SVM: support vector machine
XGBoost: eXtreme gradient boosting
