# Supplementary material for: Use of Multiprognostic Index Domain Scores, Clinical Data, and Machine Learning to Improve 12-Month Mortality Risk Prediction in Older Hospitalized Patients: Prospective Cohort Study
Source: J Med Internet Res. 2021 Jun 21;23(6):e26139. doi: 10.2196/26139 (PMC8277374; doi:10.2196/26139)
Supplement: Multimedia Appendix 1 [file jmir_v23i6e26139_app1.docx]

## The Machine Learning algorithms

*Logistic Regression* (LR) is a widely used classification algorithm which can be implemented with and without regularisation. The latter is a penalty against model complexity, in which, the estimated regression coefficients are shrunk towards zero to reduce variance (poor fit in the validation dataset) and improve generalisability and prediction accuracy at the expense of an increase in bias (increased error in the training dataset). The strength of the regularisation is controlled by the hyperparameter, lambda (λ). In this study we used logistic regression without regularisation to provide results for a standard logistic regression model, and also added an “L2” penalty (the square of the magnitude of the coefficients) to provide results based on what is known as “*Ridge Regression*”. A *Decision Tree* (DT) classifier consists of a hierarchy of decision nodes/feature thresholds which start with the “trunk” of the tree in which the most predictive feature is used to split the data and which culminate in leaf nodes in which each sample is assigned a class label. DT’s are intuitive and easily interpretative and feature Interactions are dealt with implicitly. However, small changes in data cause different tree structures and their increased complexity can increase variance. A *Random forest* (RF) is a collection of multiple classification trees where each tree casts a unit vote for the most popular class and then, combining these results, the final classification is obtained. The trees are formed using a random sample of both the data and the available features. The technique has high accuracy in classification, tolerates outliers, noise and does not over-adjust. The *Support Vector Machine* (SVM) constructs a linear-decision surface over the features which are assigned non-linearly to a high dimension space. The distance between the data points closest to the separating hyperplane is maximized. *eXtreme Gradient Boosting* (XGBoost) builds an ensemble of decision trees by iteratively focusing on harder to predict subsets of the training data. The *Naïve Bayes* classifier is a fast, probabilistic algorithm based on The Bayes Theorem for predicting conditional probabilities. It is so-called” Naïve” on the assumption that all features are independent. Since this is generally not the case, performance can be affected. *Neural Networks* are inspired by the structure and functional aspects of biological neural networks. They consist of an interconnected group of artificial neurons and adapt their structure based on information flowing through the network during the learning phase. Connections among units are optimized until the prediction error is minimized. *K-Nearest Neighbours* (kNN) is a fast, non-parametric method that classifies unlabelled observations by assigning them to the class of its k nearest neighbouring samples. Distances between all samples are calculated and the kNN are chosen. The value of k significantly impacts performance; when k is one, the bias will be zero, whereas large k reduces variance caused by random error but increases bias.
