# Supplementary material for: Use of Multiprognostic Index Domain Scores, Clinical Data, and Machine Learning to Improve 12-Month Mortality Risk Prediction in Older Hospitalized Patients: Prospective Cohort Study
Source: J Med Internet Res. 2021 Jun 21;23(6):e26139. doi: 10.2196/26139 (PMC8277374; doi:10.2196/26139)
Supplement: Multimedia Appendix 3 [file jmir_v23i6e26139_app3.pdf]

In [1]:
```
import pandas as pd
import numpy as np
import matplotlib.pyplot as plt
%matplotlib inline
import seaborn as sn
```

In [2]:
```
#Change working directory
import os
os.chdir("C:\\Users\\wood0454\\Documents\\PythonProjects\\MPI")
print("Current Working Directory " , os.getcwd())
```

```
Current Working Directory  C:\Users\wood0454\Documents\PythonProjects\MPI
```

In [3]:
```
#Import the data from Stata
df=pd.DataFrame(pd.read_excel('MPI4.xlsx'))
df=pd.get_dummies(df, columns=["Cohabitationstatus"], prefix=["Cohab"])
```

In [4]:
```
df['Urea/Cr']=df['UreammolL']/df['CreatinineumolL']
```

In [5]:
```
#Impute missing values
df=df.fillna(df.mean())

print(df.isnull().sum().sum())
```

```
0
```

In [6]:
```
#Create the features dataframe
x = df.loc[:, df.columns != 'twelveMonth_Death']
```

In [7]:
```
x.columns
```

Out[7]:
```
Index(['Age', 'ADLscore', 'IADLscore', 'SPMSQscore', 'ESSscore',
       'CIRS_ISscore', 'BMI', 'MNAscore', 'SodiummmolL', 'UreammolL',
       'CreatinineumolL', 'AlbumingL', 'HaemoglobingL',
       'TotalnoofmedicationsRegular', 'ARSscore', 'eGFR_num', 'CRP_num',
       'gender', 'Cohab_1', 'Cohab_2', 'Cohab_3', 'Urea/Cr'],
      dtype='object')
```

In [8]:
```
x.columns = ['Age', 'ADL', 'IADL',
       'SPMSQ', 'ESS', 'CIRS', 'BMI','MNA', 'Sodium','Urea','Creat',
       'Albumin', 'Hgb', 'No.Meds', 'ARS','eGFR',
       'CRP', 'gender','Cohab1', 'Cohab2', 'Cohab3','Urea/Cr']
```

In [9]:
```
# get correlations
corrMatrix = x.corr(method='spearman')
```

In [10]:
```
corrMatrix.shape
```

Out[10]:
```
(22, 22)
```

In [13]:
```
fig, ax = plt.subplots(figsize=(12, 10))
plt.style.use('ggplot')

# mask
mask = np.triu(np.ones_like(corrMatrix, dtype=np.bool))
# adjust mask and df
mask = mask[1:, :-1]
corr = corrMatrix.iloc[1:,:-1].copy()
# color map
cmap = sn.color_palette("coolwarm", as_cmap=True)
# plot heatmap
sn.heatmap(corr, mask=mask, annot=True, fmt=".2f",
           linewidths=5, cmap=cmap, vmin=-1, vmax=1,
           cbar_kws={"shrink": .8}, square=True)
# ticks
yticks = [i.upper() for i in corr.index]
xticks = [i.upper() for i in corr.columns]
plt.yticks(plt.yticks()[0], labels=yticks, rotation=0)
plt.xticks(plt.xticks()[0], labels=xticks)
# title
title = ''
plt.title(title, loc='left', fontsize=18)
plt.savefig('CorrelationMatrix.png',format='png',dpi=100)
plt.show()
```

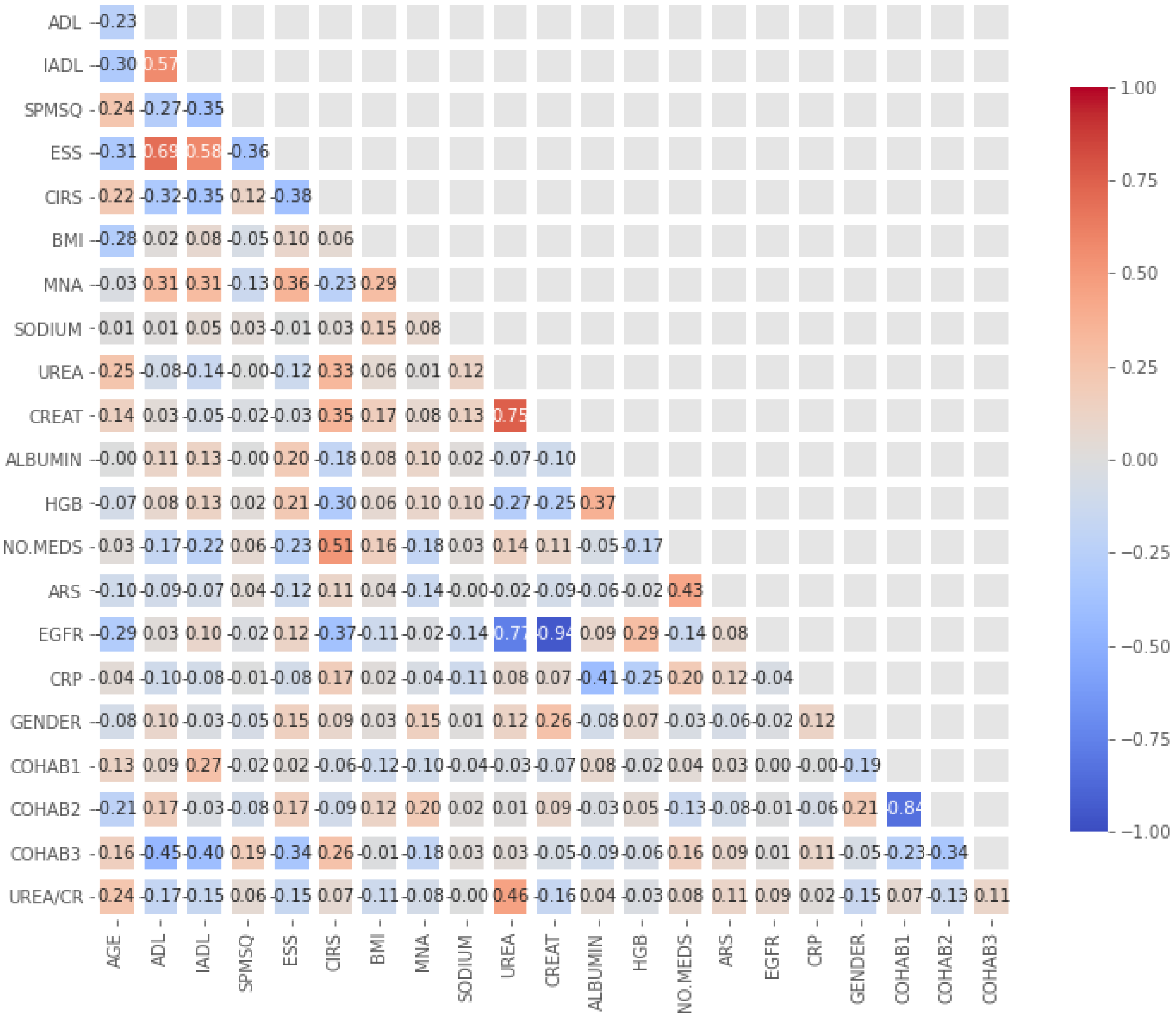

In [ ]:
