# Supplementary material for: Use of Multiprognostic Index Domain Scores, Clinical Data, and Machine Learning to Improve 12-Month Mortality Risk Prediction in Older Hospitalized Patients: Prospective Cohort Study
Source: J Med Internet Res. 2021 Jun 21;23(6):e26139. doi: 10.2196/26139 (PMC8277374; doi:10.2196/26139)
Supplement: Multimedia Appendix 4 [file jmir_v23i6e26139_app4.pdf]

In [ ]:

```
##########################################################
#Feature Importance data for XGBoost using Feature-set 1
##########################################################
importance1=pd.DataFrame(
    {
        'feature_names': x.columns,
        'feature_importances': xgbbest_clf.feature_importances_
    }
).sort_values(
    'feature_importances', ascending=True
)

####################################
#Export the feature importance data
####################################
importance1.to_excel("importance1.xlsx")
```
