# Supplementary material for: Use of Multiprognostic Index Domain Scores, Clinical Data, and Machine Learning to Improve 12-Month Mortality Risk Prediction in Older Hospitalized Patients: Prospective Cohort Study
Source: J Med Internet Res. 2021 Jun 21;23(6):e26139. doi: 10.2196/26139 (PMC8277374; doi:10.2196/26139)
Supplement: Multimedia Appendix 5 [file jmir_v23i6e26139_app5.pdf]

In [ ]:
```
###################################
#ROC plot data for Feature-set 1
###################################
lr_fpr, lr_tpr, lr_threasholds=roc_curve(y_test, lr_y_pred_prob)
dt_fpr, dt_tpr, dt_threasholds=roc_curve(y_test, dt_y_pred_prob)
rf_fpr, rf_tpr, rf_threasholds=roc_curve(y_test, rf_y_pred_prob)
xgb_fpr, xgb_tpr, xgb_threasholds=roc_curve(y_test, xgb_y_pred_prob)
knn_fpr, knn_tpr, knn_threasholds=roc_curve(y_test, knn_y_pred_prob)
nn_fpr, nn_tpr, nn_threasholds=roc_curve(y_test, nn_y_pred_prob)
svm_fpr, svm_tpr, svm_threasholds=roc_curve(y_test, svm_y_pred_prob)
LR_fpr, LR_tpr, LR_threasholds=roc_curve(y_test, LR_y_pred_prob[:,1])
nb_fpr, nb_tpr, nb_threasholds=roc_curve(y_test, nb_y_pred_prob[:,1])

roc1=pd.DataFrame([lr_fpr,lr_tpr,dt_fpr,dt_tpr,rf_fpr, rf_tpr,xgb_fpr,
                   xgb_tpr,knn_fpr,knn_tpr,nn_fpr,nn_tpr,svm_fpr,svm_tpr,
                  LR_fpr, LR_tpr,nb_fpr, nb_tpr]).T
roc1.columns=('lr_fpr','lr_tpr','dt_fpr','dt_tpr','rf_fpr','rf_tpr',
              'xgb_fpr','xgb_tpr','knn_fpr','knn_tpr','nn_fpr','nn_tpr',
             'svm_fpr','svm_tpr','LR_fpr','LR_tpr','nb_fpr','nb_tpr')

auc1=pd.DataFrame([dt_roc_auc,knn_roc_auc,LR_roc_auc,svm_roc_auc,
                   nb_roc_auc,lr_roc_auc,nn_roc_auc,rf_roc_auc,xgb_roc_auc]).T
auc1.columns=('dt_roc_auc','knn_roc_auc','LR_roc_auc','svm_roc_auc',
              'nb_roc_auc','lr_roc_auc','nn_roc_auc','rf_roc_auc','xgb_roc_au
c')

####################################
#Export the ROC data
####################################
roc1.to_excel("roc1.xlsx")
auc1.to_excel("auc1.xlsx")
```
