# Supplementary material for: Use of Multiprognostic Index Domain Scores, Clinical Data, and Machine Learning to Improve 12-Month Mortality Risk Prediction in Older Hospitalized Patients: Prospective Cohort Study
Source: J Med Internet Res. 2021 Jun 21;23(6):e26139. doi: 10.2196/26139 (PMC8277374; doi:10.2196/26139)
Supplement: Multimedia Appendix 6 [file jmir_v23i6e26139_app6.pdf]

In [12]:
```
#pip install seaborn
```

In [13]:
```
import seaborn as sns
sns.__version__
```

Out[13]:
```
'0.11.0'
```

In [14]:
```
import matplotlib.pyplot as plt
import numpy as np
import pandas as pd
```

In [15]:
```
#Import the data
import os
os.chdir("C:\\Users\\wood0454\\Documents\\PythonProjects\\MPI")
df=pd.DataFrame(pd.read_excel('MPI4.xlsx'))
df.columns
```

Out[15]:
```
Index(['Age', 'Cohabitationstatus', 'ADLscore', 'IADLscore', 'SPMSQscore',
       'ESSscore', 'CIRS_ISscore', 'BMI', 'MNAscore', 'SodiummmolL',
       'UreammolL', 'CreatinineumolL', 'AlbumingL', 'HaemoglobingL',
       'TotalnoofmedicationsRegular', 'ARSscore', 'twelveMonth_Death',
       'eGFR_num', 'CRP_num', 'gender'],
      dtype='object')
```

In [16]:
```
data = df[['Age','MNAscore','IADLscore','CIRS_ISscore','twelveMonth_Death']]
data.head()
```

Out[16]:

	Age	MNAscore	IADLscore	CIRS_ISscore	twelveMonth_Death
**0**	81	20.0	7	1.769231	0
**1**	90	20.0	8	1.615385	0
**2**	84	23.5	4	2.538462	0
**3**	76	8.5	6	2.692308	0
**4**	86	22.5	3	2.769231	1

In [17]:
```
import seaborn as sns
sns.set_theme(style="whitegrid")

fig, axs = plt.subplots(2, 2,  figsize=(20,10))

#fig, axs = plt.subplots(ncols=2, nrows=2,  figsize=(20,10))

# Make a dictionary with one specific color per group:
my_pal = {1: "silver", 0: "whitesmoke"}

ax1=sns.violinplot(x="twelveMonth_Death", y="MNAscore",
                    data=df, palette=my_pal, split=True,
                    scale="count",ax=axs[0,0])
ax1.set_title('MNA', fontsize=16);
ax1.set_xlabel('');
ax1.set_ylabel('MNA score', fontsize=16);

ax2 = sns.violinplot(x='twelveMonth_Death', y="IADLscore",
                    data=df,  split=True,
                    scale="count",  height=10, aspect=1,ax=axs[0,1],palette=my
_pal)
ax2.set_title('IADL', fontsize=16);
ax2.set_xlabel('');
ax2.set_ylabel('IADL score', fontsize=16);

ax3= sns.violinplot(x='twelveMonth_Death', y="CIRS_ISscore",
                    data=df, palette=my_pal, split=True,
                    scale="count",  height=10, aspect=1,ax=axs[1,0])
ax3.set_title('CIRS score', fontsize=16);
ax3.set_xlabel('12-month mortality status',fontsize=16);
ax3.set_ylabel('CIRS score', fontsize=16);

ax4 = sns.violinplot(x='twelveMonth_Death', y="UreammolL",
                    data=df, palette=my_pal, split=False,
                    scale="count",  height=10, aspect=1,ax=axs[1,1])
ax4.set_title('Urea', fontsize=16);
ax4.set_xlabel('12-month mortality status',fontsize=16);
ax4.set_ylabel('Urea', fontsize=16);

for ax in axs.flat:
    labels = [item.get_text() for item in ax.get_xticklabels()]
    labels[0] = 'Alive'
    labels[1] = 'Deceased'
    ax.set_xticklabels(labels, fontsize=16)

plt.savefig('ViolinPlts.png', dpi=60)
```

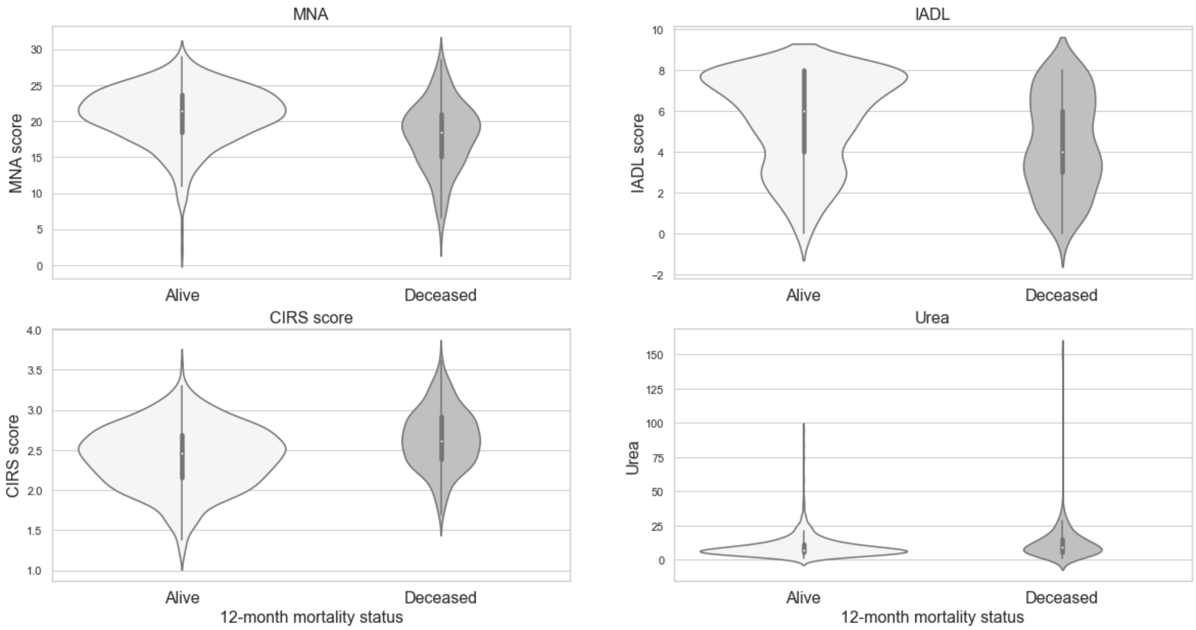
MNA
MNA score
30
25
20
15
10
5
0
Alive
Deceased
IADL
IADL score
10
8
6
4
2
0
−2
Alive
Deceased
CIRS score
CIRS score
4.0
3.5
3.0
2.5
2.0
1.5
1.0
Alive
Deceased
12-month mortality status
Urea
Urea
150
125
100
75
50
25
0
Alive
Deceased
12-month mortality status
