# Supplementary material for: Use of Multiprognostic Index Domain Scores, Clinical Data, and Machine Learning to Improve 12-Month Mortality Risk Prediction in Older Hospitalized Patients: Prospective Cohort Study
Source: J Med Internet Res. 2021 Jun 21;23(6):e26139. doi: 10.2196/26139 (PMC8277374; doi:10.2196/26139)

In [2]:
```
import pandas as pd
import numpy as np
%matplotlib inline
import matplotlib.pyplot as plt

from sklearn.preprocessing import MinMaxScaler
from sklearn.model_selection import KFold
from sklearn.model_selection import cross_validate,  cross_val_score, cross_va
l_predict

from sklearn.linear_model import LogisticRegression
from sklearn.tree import DecisionTreeClassifier
from sklearn.svm import SVC
import xgboost as xgb
from xgboost.sklearn import XGBClassifier
from sklearn.ensemble import RandomForestClassifier
from sklearn.neighbors import KNeighborsClassifier

from keras.models import Sequential
from keras.layers import Dense
from keras.wrappers.scikit_learn import KerasClassifier

from sklearn import metrics
from sklearn.metrics import f1_score,precision_score,recall_score,roc_auc_scor
e,accuracy_score,roc_curve, confusion_matrix
```

In [3]:
```
#Change working directory
import os
os.chdir("C:\\Users\\wood0454\\Documents\\PythonProjects\\MPI")
print("Current Working Directory " , os.getcwd())
```

```
Current Working Directory  C:\Users\wood0454\Documents\PythonProjects\MPI
```

In [86]:
```
#Import the data from Excel
df=pd.DataFrame(pd.read_excel('MPI4.xlsx'))
df=pd.get_dummies(df, columns=["Cohabitationstatus"], prefix=["Cohab"])
```

In [87]:
```
df.head()
```

Out[87]:

	Age	ADLscore	IADLscore	SPMSQscore	ESSscore	CIRS_ISscore	BMI	MNAscore	Sc
**0**	81	6	7	2	20	1.769231	22.913033	20.0	
**1**	90	6	8	0	20	1.615385	28.515625	20.0	
**2**	84	6	4	4	16	2.538462	26.971815	23.5	
**3**	76	6	6	2	18	2.692308	16.666667	8.5	
**4**	86	5	3	1	13	2.769231	28.875440	22.5	

5 rows × 22 columns

In [88]:
```python
#Impute missing values
df=df.fillna(df.mean())
```

In [89]:
```python
df['Urea/Cr']=df['UreammolL']/df['CreatinineumolL']
```

In [90]:
```python
#Seperate the feature variables (x) and the target variable (y)
x = df.loc[:, df.columns != 'twelveMonth_Death',]
y = df['twelveMonth_Death']
```

In [91]:
```python
x.head()
```

Out[91]:

	Age	ADLscore	IADLscore	SPMSQscore	ESSscore	CIRS_ISscore	BMI	MNAscore	Sc
**0**	81	6	7	2	20	1.769231	22.913033	20.0	
**1**	90	6	8	0	20	1.615385	28.515625	20.0	
**2**	84	6	4	4	16	2.538462	26.971815	23.5	
**3**	76	6	6	2	18	2.692308	16.666667	8.5	
**4**	86	5	3	1	13	2.769231	28.875440	22.5	

5 rows × 22 columns

In [ ]:
```python

```

In [10]:
```python
#Rename the features
x.columns = ['Age', 'ADL', 'IADL',
       'SPMSQ', 'ESS', 'CIRS', 'BMI','MNA', 'Sodium','Urea','Creat',
       'Albumin', 'Hgb', 'No.Meds', 'ARS','eGFR',
       'CRP', 'Gender','Cohab1', 'Cohab2','Cohab3','Urea/Cr']
```

In [11]:
```python
x.head()
```

Out[11]:

	Age	ADL	IADL	SPMSQ	ESS	CIRS	BMI	MNA	Sodium	Urea	...	Hgb	No.Mec
**0**	81	6	7	2	20	1.769231	22.913033	20.0	135.0	7.5	...	136.0	
**1**	90	6	8	0	20	1.615385	28.515625	20.0	133.0	7.2	...	130.0	1
**2**	84	6	4	4	16	2.538462	26.971815	23.5	132.0	6.3	...	101.0	1
**3**	76	6	6	2	18	2.692308	16.666667	8.5	139.0	5.1	...	125.0	1
**4**	86	5	3	1	13	2.769231	28.875440	22.5	138.0	25.4	...	104.0	1

5 rows × 22 columns

In [12]:
```
features = x[x.columns]

#Scale the x features
from sklearn.compose import ColumnTransformer
from sklearn.preprocessing import StandardScaler

ct = ColumnTransformer([
        ('somename', StandardScaler(), ['Age', 'ADL', 'IADL',
       'SPMSQ', 'ESS', 'CIRS', 'BMI','MNA', 'Sodium','Urea','Creat',
       'Albumin', 'Hgb', 'No.Meds', 'ARS','eGFR',
       'CRP', 'Urea/Cr'])
    ], remainder='passthrough')

xnew= pd.DataFrame(ct.fit_transform(features),columns=x.columns)
x=xnew
```

In [13]:
```
########################################
#Split Data for Training and Validation
########################################
from sklearn.model_selection import train_test_split
x_train, x_test, y_train, y_test = train_test_split(x,y, test_size=0.3, random
_state=13)
print(x_train.shape[0], x_test.shape[0])
print(x_train.shape[1])
```

```
515 222
22
```

In [14]:
```
##############################################################################
#Training dataset accuracy with 10-fold cross-validation using Grid Search
##############################################################################
```

In [15]:
```
########################################
#Logistic Regression - Ridge Regression
########################################
```

In [16]:
```
from sklearn.linear_model import LogisticRegression
from sklearn.model_selection import GridSearchCV
lr=LogisticRegression()
param_grid = {
         'penalty' : ['l2'],
         'C' : np.logspace(-4, 4,100),
        'solver': ['liblinear', 'newton-cg', 'lbfgs', 'sag', 'saga'],
       'max_iter':[100000]
        }

# Grid search object
grid_search = GridSearchCV(estimator=lr,param_grid = param_grid, cv = 10, verb
ose=True)

# Fit the grid search to the data
grid_search.fit(x_train, y_train)
```

```
Fitting 10 folds for each of 500 candidates, totalling 5000 fits

[Parallel(n_jobs=1)]: Using backend SequentialBackend with 1 concurrent worke
rs.
[Parallel(n_jobs=1)]: Done 5000 out of 5000 | elapsed:  3.1min finished
```

Out[16]:
```
GridSearchCV(cv=10, estimator=LogisticRegression(),
             param_grid={'C': array([1.00000000e-04, 1.20450354e-04, 1.450828
78e-04, 1.74752840e-04,
       2.10490414e-04, 2.53536449e-04, 3.05385551e-04, 3.67837977e-04,
       4.43062146e-04, 5.33669923e-04, 6.42807312e-04, 7.74263683e-04,
       9.32603347e-04, 1.12332403e-03, 1.35304777e-03, 1.62975083e-03,
       1.96304065e-03, 2.36448941e-03, 2.84803...
       2.91505306e+02, 3.51119173e+02, 4.22924287e+02, 5.09413801e+02,
       6.13590727e+02, 7.39072203e+02, 8.90215085e+02, 1.07226722e+03,
       1.29154967e+03, 1.55567614e+03, 1.87381742e+03, 2.25701972e+03,
       2.71858824e+03, 3.27454916e+03, 3.94420606e+03, 4.75081016e+03,
       5.72236766e+03, 6.89261210e+03, 8.30217568e+03, 1.00000000e+04]),
                         'max_iter': [100000], 'penalty': ['l2'],
                         'solver': ['liblinear', 'newton-cg', 'lbfgs', 'sag',
                                    'saga']},
             verbose=True)
```

In [17]:
```
grid_search.best_estimator_
```

Out[17]:
```
LogisticRegression(C=0.022051307399030457, max_iter=100000, solver='newton-c
g')
```

In [18]:
```
#Fit the regressoor to the training set and make predictions on  the test set
lrbest_clf=grid_search.best_estimator_.fit(x_train,y_train)

lr_y_pred_prob=lrbest_clf.predict_proba(x_test)[:,1]
lr_y_pred=lrbest_clf.predict(x_test)

lr_roc_auc = roc_auc_score(y_test, lr_y_pred_prob).round(4)
lr_accuracy=accuracy_score(y_test, lr_y_pred).round(4)
lr_f1score = f1_score(y_test, lr_y_pred, average='binary')
lr_precision = precision_score(y_test, lr_y_pred, labels=[1,2], average='micr
o')
lr_recall = recall_score(y_test, lr_y_pred, labels=[1,2], average='micro')

print("lr_accuracy_score:%.3f, lr_preci_score:%.3f, lr_recall_score:%.3f, lr_f
1_score:%.3f, lr_auc:%.3f"
%(lr_accuracy, lr_precision, lr_recall, lr_f1score, lr_roc_auc))
```

```
lr_accuracy_score:0.743, lr_preci_score:0.640, lr_recall_score:0.250, lr_f1_s
core:0.360, lr_auc:0.749
```

In [19]:
```
#Data for ROC plot
lr_fpr, lr_tpr, lr_threasholds=roc_curve(y_test, lr_y_pred_prob)
```

In [20]:
```
##########################################
#Decision Tree
##########################################
```

In [21]:
```
from sklearn.tree import DecisionTreeClassifier, plot_tree, export_text
from sklearn.model_selection import GridSearchCV

dt = DecisionTreeClassifier()
param_grid = {
          'criterion':['entropy'],
          'max_depth':[3,4,6],
          'max_leaf_nodes': [2,10,1],
          'min_samples_split': [10],
           'max_features':[None], 'max_leaf_nodes':[None],
            'min_impurity_decrease':[0.0], 'min_impurity_split':[None],
            'min_samples_leaf':[1,2,5],
            'min_weight_fraction_leaf':[0.02,0.03], 'random_state':[None],
            'splitter':['best']}
# Grid search object
grid_search = GridSearchCV(estimator=dt,param_grid = param_grid, cv = 10, verb
ose=1)

# Fit the grid search to the data
grid_search.fit(x_train, y_train)
```

```
Fitting 10 folds for each of 18 candidates, totalling 180 fits

[Parallel(n_jobs=1)]: Using backend SequentialBackend with 1 concurrent worke
rs.
[Parallel(n_jobs=1)]: Done 180 out of 180 | elapsed:    1.7s finished
```

Out[21]:
```
GridSearchCV(cv=10, estimator=DecisionTreeClassifier(),
             param_grid={'criterion': ['entropy'], 'max_depth': [3, 4, 6],
                         'max_features': [None], 'max_leaf_nodes': [None],
                         'min_impurity_decrease': [0.0],
                         'min_impurity_split': [None],
                         'min_samples_leaf': [1, 2, 5],
                         'min_samples_split': [10],
                         'min_weight_fraction_leaf': [0.02, 0.03],
                         'random_state': [None], 'splitter': ['best']},
             verbose=1)
```

In [22]:
```
grid_search.best_estimator_
```

Out[22]:
```
DecisionTreeClassifier(criterion='entropy', max_depth=3, min_samples_split=1
0,
                       min_weight_fraction_leaf=0.02)
```

In [23]:
```
#Fit the regressor to the training set and make predictions on  the test set
dtbest_clf=grid_search.best_estimator_.fit(x_train,y_train)

dt_y_pred_prob=dtbest_clf.predict_proba(x_test)[:,1]
dt_y_pred=dtbest_clf.predict(x_test)

dt_roc_auc = roc_auc_score(y_test, dt_y_pred_prob).round(4)
dt_accuracy=accuracy_score(y_test, dt_y_pred).round(4)
dt_f1score = f1_score(y_test, dt_y_pred, average='binary')
dt_precision = precision_score(y_test, dt_y_pred, labels=[1,2], average='micr
o')
dt_recall = recall_score(y_test, dt_y_pred, labels=[1,2], average='micro')

print("dt_accuracy_score:%.3f, dt_preci_score:%.3f, dt_recall_score:%.3f, dt_f
1_score:%.3f, dt_auc:%.3f"
%(dt_accuracy, dt_precision, dt_recall, dt_f1score, dt_roc_auc))
```

```
dt_accuracy_score:0.712, dt_preci_score:0.500, dt_recall_score:0.031, dt_f1_s
core:0.059, dt_auc:0.689
```

In [24]:
```
#Data for ROC plot
dt_fpr, dt_tpr, dt_threasholds=roc_curve(y_test, dt_y_pred_prob)
```

In [25]:
```
##############################################
#Random Forest
##############################################
```

In [26]:
```
from sklearn.ensemble import RandomForestClassifier
from sklearn.model_selection import GridSearchCV

param_grid = {
    'bootstrap': [True],
    'max_depth': [3, 4, 5,6],
    'max_features': list(range(6,11,1)),
    'min_samples_leaf': [3, 4, 5],
    'min_samples_split': [8, 10, 12],
    'n_estimators': list(range(10,101,10))
        }

rf=RandomForestClassifier()
# Grid search object
grid_search = GridSearchCV(estimator=rf,param_grid = param_grid, cv = 10, verb
ose=1)

# Fit the grid search to the data
rfbest_clf=grid_search.fit(x_train, y_train)
```

```
[Parallel(n_jobs=1)]: Using backend SequentialBackend with 1 concurrent worke
rs.

Fitting 10 folds for each of 1800 candidates, totalling 18000 fits

[Parallel(n_jobs=1)]: Done 18000 out of 18000 | elapsed: 33.7min finished
```

In [27]:
```
grid_search.best_estimator_
```

Out[27]:
```
RandomForestClassifier(max_depth=6, max_features=7, min_samples_leaf=3,
                       min_samples_split=10, n_estimators=60)
```

In [28]:
```
#Mmake predictions on  the test set
rf_y_pred_prob=rfbest_clf.predict_proba(x_test)[:,1]
rf_y_pred=rfbest_clf.predict(x_test)

rf_roc_auc = roc_auc_score(y_test, rf_y_pred_prob).round(4)
rf_accuracy=accuracy_score(y_test, rf_y_pred).round(4)
rf_f1score = f1_score(y_test, rf_y_pred, average='binary')
rf_precision = precision_score(y_test, rf_y_pred, labels=[1,2], average='micr
o')
rf_recall = recall_score(y_test, rf_y_pred, labels=[1,2], average='micro')

print("rf_accuracy_score:%.3f, rf_preci_score:%.3f, rf_recall_score:%.3f, rf_f
1_score:%.3f, rf_auc:%.3f"
%(rf_accuracy, rf_precision, rf_recall, rf_f1score, rf_roc_auc))
```

```
rf_accuracy_score:0.739, rf_preci_score:0.650, rf_recall_score:0.203, rf_f1_s
core:0.310, rf_auc:0.768
```

In [29]:
```
#Data for ROC plot
rf_fpr, rf_tpr, rf_threasholds=roc_curve(y_test, rf_y_pred_prob)
```

In [30]:
```
###############################################
#XGB
###############################################
```

In [31]:

```
xgb = XGBClassifier()
params = {'colsample_bytree': [0.3, 0.4],'learning_rate': [0.1, 0.01, 0.05],
                'max_depth': range (3, 6, 1), 'alpha': [1,5,10],'gamma':[0.1,
0.2, 0.3,  0.4]}

# Grid search object
grid_search = GridSearchCV(estimator=xgb,param_grid = params, cv = 10, verbose
=True, scoring='roc_auc')

# Fit the grid search to the data
grid_search.fit(x_train, y_train)
```

```
Fitting 10 folds for each of 216 candidates, totalling 2160 fits

[Parallel(n_jobs=1)]: Using backend SequentialBackend with 1 concurrent worke
rs.
[Parallel(n_jobs=1)]: Done 2160 out of 2160 | elapsed:  1.7min finished
```

Out[31]:

```
GridSearchCV(cv=10,
             estimator=XGBClassifier(base_score=None, booster=None,
                                     colsample_bylevel=None,
                                     colsample_bynode=None,
                                     colsample_bytree=None, gamma=None,
                                     gpu_id=None, importance_type='gain',
                                     interaction_constraints=None,
                                     learning_rate=None, max_delta_step=None,
                                     max_depth=None, min_child_weight=None,
                                     missing=nan, monotone_constraints=None,
                                     n_estimators=100, n_jobs=None,
                                     num_parallel_tree=None, random_state=Non
e,
                                     reg_alpha=None, reg_lambda=None,
                                     scale_pos_weight=None, subsample=None,
                                     tree_method=None, validate_parameters=No
ne,
                                     verbosity=None),
             param_grid={'alpha': [1, 5, 10], 'colsample_bytree': [0.3, 0.4],
                         'gamma': [0.1, 0.2, 0.3, 0.4],
                         'learning_rate': [0.1, 0.01, 0.05],
                         'max_depth': range(3, 6)},
             scoring='roc_auc', verbose=True)
```

In [32]:

```
grid_search.best_estimator_
```

Out[32]:

```
XGBClassifier(alpha=5, base_score=0.5, booster='gbtree', colsample_bylevel=1,
              colsample_bynode=1, colsample_bytree=0.4, gamma=0.3, gpu_id=-1,
              importance_type='gain', interaction_constraints='',
              learning_rate=0.05, max_delta_step=0, max_depth=3,
              min_child_weight=1, missing=nan, monotone_constraints='()',
              n_estimators=100, n_jobs=0, num_parallel_tree=1, random_state=
0,
              reg_alpha=5, reg_lambda=1, scale_pos_weight=1, subsample=1,
              tree_method='exact', validate_parameters=1, verbosity=None)
```

In [33]:
```
#Fit the regressoor to the training set and make predictions on  the test set
xgbbest_clf=grid_search.best_estimator_.fit(x_train,y_train)

xgb_y_pred_prob=xgbbest_clf.predict_proba(x_test)[:,1]
xgb_y_pred=xgbbest_clf.predict(x_test)

xgb_roc_auc = roc_auc_score(y_test, xgb_y_pred_prob).round(4)
xgb_accuracy=accuracy_score(y_test, xgb_y_pred).round(4)
xgb_f1score = f1_score(y_test, xgb_y_pred, average='binary')
xgb_precision = precision_score(y_test, xgb_y_pred, labels=[1,2], average='mic
ro')
xgb_recall = recall_score(y_test, xgb_y_pred, labels=[1,2], average='micro')

print("xgb_accuracy_score:%.3f, xgb_preci_score:%.3f, xgb_recall_score:%.3f, x
gb_f1_score:%.3f, xgb_auc:%.3f"
%(xgb_accuracy, xgb_precision, xgb_recall, xgb_f1score, xgb_roc_auc))
```

```
xgb_accuracy_score:0.739, xgb_preci_score:0.625, xgb_recall_score:0.234, xgb_
f1_score:0.341, xgb_auc:0.757
```

In [34]:
```
#Data for ROC plot
xgb_fpr, xgb_tpr, xgb_threasholds=roc_curve(y_test, xgb_y_pred_prob)
```

In [35]:

```
#Feature Importance
pd.DataFrame(
    {
        'feature_names': x_train.columns,
        'feature_importances': xgbbest_clf.feature_importances_
    }
).sort_values(
    'feature_importances', ascending=False
).set_index(
    'feature_names'
)
```

Out[35]:

feature_names	feature_importances
MNA	0.161858
IADL	0.101881
CIRS	0.074645
Cohab1	0.067602
Urea	0.061430
ESS	0.059577
SPMSQ	0.050119
ADL	0.047699
Creat	0.046531
Albumin	0.044062
BMI	0.041218
Age	0.038746
CRP	0.032359
Hgb	0.030685
eGFR	0.030547
Gender	0.026509
ARS	0.024034
No.Meds	0.023290
Sodium	0.020055
Urea/Cr	0.010314
Cohab2	0.006839
Cohab3	0.000000

In [36]:
```
xgbbest_clf.feature_importances_
```

Out[36]:
```
array([0.03874584, 0.04769925, 0.10188083, 0.05011909, 0.05957662,
       0.07464512, 0.04121818, 0.1618582 , 0.02005476, 0.06142959,
       0.04653054, 0.04406214, 0.03068539, 0.02328981, 0.02403391,
       0.03054729, 0.03235868, 0.02650893, 0.06760213, 0.00683932,
       0.        , 0.01031436], dtype=float32)
```

In [37]:
```
import matplotlib.pyplot as plt
%matplotlib inline
feat_importances = pd.Series(xgbbest_clf.feature_importances_, index=x_train.c
olumns)
feat_importances.nsmallest(22).plot(kind='barh')
plt.title("XGBoost feature importance", fontsize= 12)
plt.xlabel('Relative importance', fontsize= 10)
plt.tight_layout()
plt.savefig("XGB_FI Test Data4.png",format='png',dpi=2400)
plt.show()
```

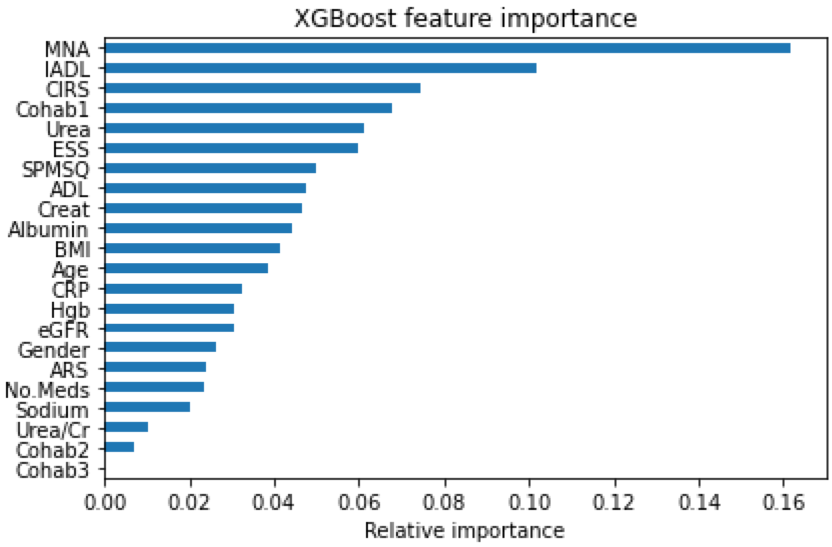

In [38]:
```
x.columns = ['Age', 'ADL', 'IADL',
       'SPMSQ', 'ESS', 'CIRS', 'BMI','MNA', 'Sodium','Urea','Creat',
       'Albumin', 'Hgb', 'No.Meds', 'ARS','eGFR',
       'CRP', 'Gender','Cohab1', 'Cohab2','Cohab3','Urea/Cr']
```

In [39]:
```
importance4=pd.DataFrame(
    {
        'feature_names': x.columns,
        'feature_importances': xgbbest_clf.feature_importances_
    }
).sort_values(
    'feature_importances', ascending=True
)
```

In [40]:
```
 xgb.importance_type
```

Out[40]:
```
'gain'
```

In [41]:
```
fig, ax = plt.subplots(figsize=(6,5))
ax = fig.gca()
plt.style.use('ggplot')

ax.set_xticks(np.arange(0, 0.18, 0.02))
ax.yaxis.grid(linestyle='dashed')
ax.xaxis.grid(False)
plt.scatter(importance4['feature_importances'],importance4['feature_names'])
ax.set_xlim(xmin=0)
plt.yticks(ha='left',position=(-0.13,0))
plt.title("XGBoost feature importance", fontsize= 15)
plt.xlabel('Relative importance', fontsize= 12)
plt.savefig("XGB_FI Test Data4_scatter.png",format='png',dpi=2400)
plt.show()
```

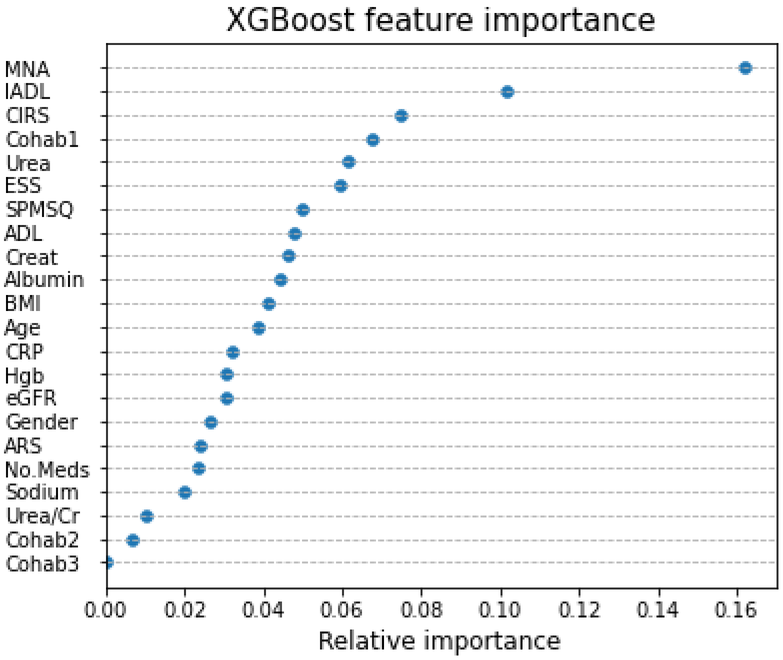

In [45]:
```
#############################################
#Combine feature plots for datasets 1 , 2, 3
#############################################
importance1=pd.read_excel('importance1.xlsx', index_col=0)
importance2=pd.read_excel('importance2.xlsx', index_col=0)
importance3=pd.read_excel('importance3.xlsx', index_col=0)
```

In [46]:
```
type(importance4)
```

Out[46]:
```
pandas.core.frame.DataFrame
```

In [53]:
```
#fig, axs = plt.subplots(ncols=1, nrows=2)
fig, axs = plt.subplots(2,2,figsize=(12,12))
plt.style.use('ggplot')

plt.subplot(2, 2, 1)
plt.xlim(xmin=0, xmax=0.28)
ax1=plt.scatter(importance1['feature_importances'],importance1['feature_names'
])
plt.title('ROC Test Feature-set 1', fontsize=16);
plt.xlabel('Relative importance', fontsize= 12);
plt.yticks(ha='left',position=(-0.13,0))
plt.grid(axis='x')

plt.subplot(2, 2, 2)
plt.xlim(xmin=0,xmax=0.16)
ax2=plt.scatter(importance2['feature_importances'],importance2['feature_names'
])
plt.title('ROC Test Feature-set 2', fontsize=16);
plt.xlabel('Relative importance', fontsize= 12);
plt.yticks(ha='left',position=(-0.13,0))
plt.grid(axis='x')

plt.subplot(2, 2, 3)
plt.xlim(xmin=0, xmax=0.28)
ax1=plt.scatter(importance3['feature_importances'],importance3['feature_names'
])
plt.title('ROC Test Feature-set 3', fontsize=16);
plt.xlabel('Relative importance', fontsize= 12);
plt.yticks(ha='left',position=(-0.13,0))
plt.grid(axis='x')

plt.subplot(2, 2, 4)
plt.xlim(xmin=0,xmax=0.18)
ax2=plt.scatter(importance4['feature_importances'],importance4['feature_names'
])
plt.title('ROC Test Feature-set 4', fontsize=16);
plt.xlabel('Relative importance', fontsize= 12);
plt.yticks(ha='left',position=(-0.13,0))
plt.grid(axis='x')

plt.savefig('XGBoost_FI.png', dpi=1200)
```

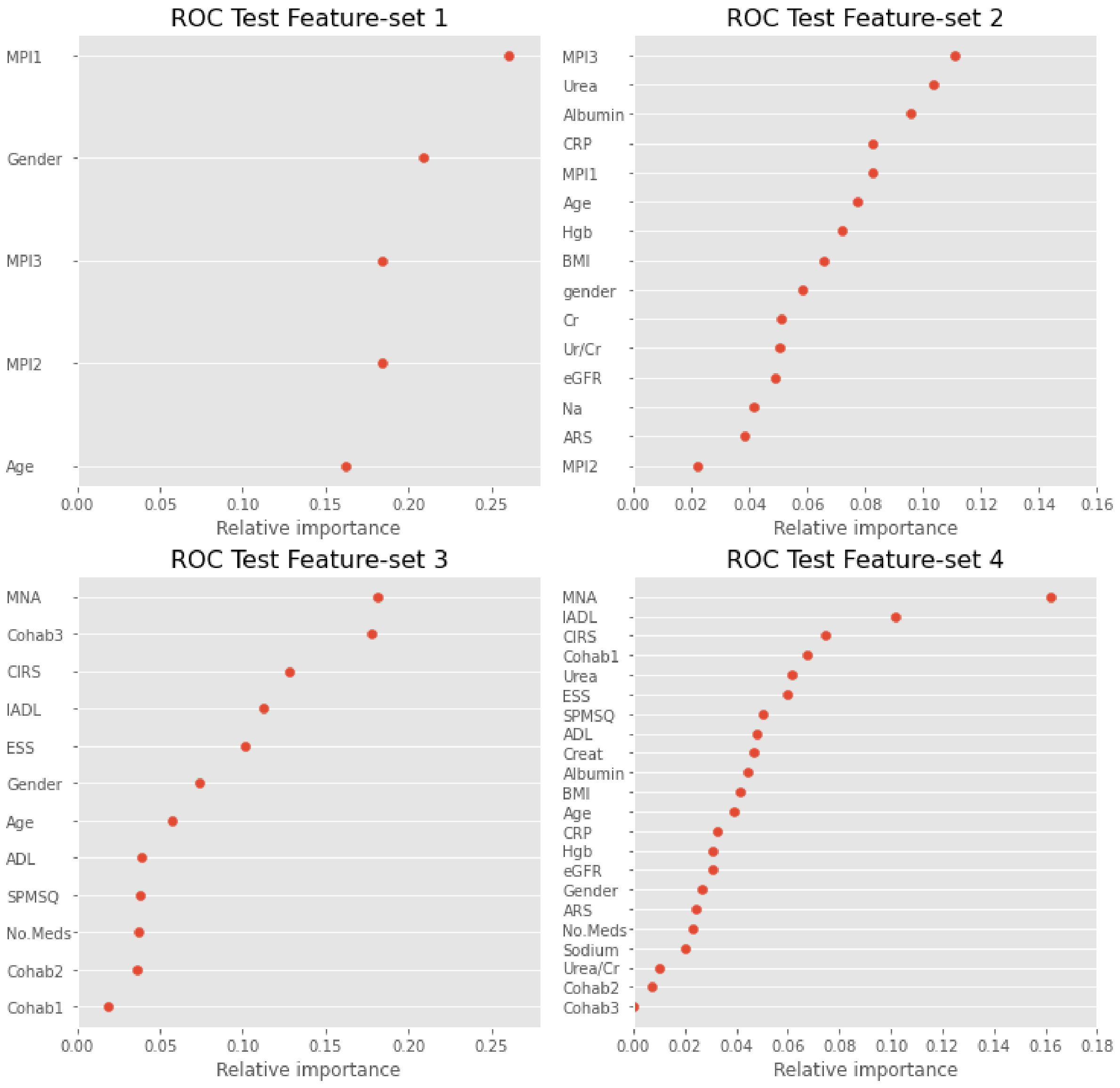
ROC Test Feature-set 1
MPI1
Gender
MPI3
MPI2
Age
0.00 0.05 0.10 0.15 0.20 0.25
Relative importance
ROC Test Feature-set 2
MPI3
Urea
Albumin
CRP
MPI1
Age
Hgb
BMI
gender
Cr
Ur/Cr
eGFR
Na
ARS
MPI2
0.00 0.02 0.04 0.06 0.08 0.10 0.12 0.14 0.16
Relative importance
ROC Test Feature-set 3
MNA
Cohab3
CIRS
IADL
ESS
Gender
Age
ADL
SPMSQ
No.Meds
Cohab2
Cohab1
0.00 0.05 0.10 0.15 0.20 0.25
Relative importance
ROC Test Feature-set 4
MNA
IADL
CIRS
Cohab1
Urea
ESS
SPMSQ
ADL
Creat
Albumin
BMI
Age
CRP
Hgb
eGFR
Gender
ARS
No.Meds
Sodium
Urea/Cr
Cohab2
Cohab3
0.00 0.02 0.04 0.06 0.08 0.10 0.12 0.14 0.16 0.18
Relative importance

In [54]:

```
################################################
#KNN
################################################
```

In [55]:
```
knn = KNeighborsClassifier()
grid_params = {
    'n_neighbors':range(1,20,1),
    'weights':['uniform','distance'],
    'metric':['minkowski','manhattan']
    }

# Grid search object
grid_search = GridSearchCV(knn,grid_params,verbose=1, cv=10, n_jobs=-1)

# Fit the grid search to the data
grid_search.fit(x_train, y_train)
```

```
Fitting 10 folds for each of 76 candidates, totalling 760 fits

[Parallel(n_jobs=-1)]: Using backend LokyBackend with 4 concurrent workers.
[Parallel(n_jobs=-1)]: Done  68 tasks      | elapsed:    3.7s
[Parallel(n_jobs=-1)]: Done 760 out of 760 | elapsed:    7.5s finished
```

Out[55]:
```
GridSearchCV(cv=10, estimator=KNeighborsClassifier(), n_jobs=-1,
             param_grid={'metric': ['minkowski', 'manhattan'],
                         'n_neighbors': range(1, 20),
                         'weights': ['uniform', 'distance']},
             verbose=1)
```

In [56]:
```
grid_search.best_estimator_
```

Out[56]:
```
KNeighborsClassifier(n_neighbors=19)
```

In [57]:
```
#Fit the regressoor to the training set and make predictions on  the test set
knnbest_clf=grid_search.best_estimator_.fit(x_train,y_train)

knn_y_pred_prob=knnbest_clf.predict_proba(x_test)[:,1]
knn_y_pred=knnbest_clf.predict(x_test)

knn_roc_auc = roc_auc_score(y_test, knn_y_pred_prob).round(4)
knn_accuracy=accuracy_score(y_test, knn_y_pred).round(4)
knn_f1score = f1_score(y_test, knn_y_pred, average='binary')
knn_precision = precision_score(y_test, knn_y_pred, labels=[1,2], average='mic
ro')
knn_recall = recall_score(y_test, knn_y_pred, labels=[1,2], average='micro')

print("knn_accuracy_score:%.3f, knn_preci_score:%.3f, knn_recall_score:%.3f, k
nn_f1_score:%.3f, knn_auc:%.3f"
%(knn_accuracy, knn_precision, knn_recall, knn_f1score, knn_roc_auc))
```

```
knn_accuracy_score:0.707, knn_preci_score:0.474, knn_recall_score:0.141, knn_
f1_score:0.217, knn_auc:0.715
```

In [58]:
```
#Data for ROC plot
knn_fpr, knn_tpr, knn_threasholds=roc_curve(y_test, knn_y_pred_prob)
```

In [59]:
```
##############################################
#Neural Network
##############################################
```

In [60]:
```
import numpy
from keras.models import Sequential
from keras.layers import Dense
from keras.wrappers.scikit_learn import KerasClassifier
# Function to create model, required for KerasClassifier
def create_model(activation='relu'):
#create model
    model = Sequential()
    model.add(Dense(12, input_dim=22, kernel_initializer='uniform', activation
=activation))
    model.add(Dense(1, kernel_initializer='uniform', activation='sigmoid'))
    # Compile model
    model.compile(loss='binary_crossentropy', optimizer='adam', metrics=['accu
racy'])
    return model
# fix random seed for reproducibility
seed = 7
numpy.random.seed(seed)

# create model
model = KerasClassifier(build_fn=create_model, epochs=100, batch_size=10, verb
ose=0)

# define the grid search parameters
activation = ['softmax', 'softplus', 'softsign', 'relu', 'tanh', 'sigmoid', 'h
ard_sigmoid', 'linear']
param_grid = dict(activation=activation)

grid_search = GridSearchCV(estimator=model, param_grid=param_grid, n_jobs=-1,
cv=3)
nnbest_clf=grid_search.fit(x_train, y_train)
# summarize results
print("Best: %f using %s" % (nnbest_clf.best_score_, nnbest_clf.best_params_))
means = nnbest_clf.cv_results_['mean_test_score']
stds = nnbest_clf.cv_results_['std_test_score']
params = nnbest_clf.cv_results_['params']
for mean, stdev, param in zip(means, stds, params):
    print("%f (%f) with: %r" % (mean, stdev, param))
```

```
Best: 0.745625 using {'activation': 'softplus'}
0.733963 (0.007836) with: {'activation': 'softmax'}
0.745625 (0.007417) with: {'activation': 'softplus'}
0.741749 (0.002465) with: {'activation': 'softsign'}
0.728127 (0.012586) with: {'activation': 'relu'}
0.737907 (0.015787) with: {'activation': 'tanh'}
0.739811 (0.002036) with: {'activation': 'sigmoid'}
0.733975 (0.007426) with: {'activation': 'hard_sigmoid'}
0.739789 (0.007823) with: {'activation': 'linear'}
```

In [61]:
```
nnbest_clf.best_params_
```

Out[61]:
```
{'activation': 'softplus'}
```

In [62]:
```
from sklearn.metrics import accuracy_score, precision_score, recall_score, f1_
score,roc_auc_score
nn_y_pred_prob=nnbest_clf.predict_proba(x_test)[:,1]
nn_y_pred=nnbest_clf.predict(x_test)
```

```
WARNING:tensorflow:From C:\Anaconda\lib\site-packages\tensorflow\python\keras
\wrappers\scikit_learn.py:264: Sequential.predict_proba (from tensorflow.pyth
on.keras.engine.sequential) is deprecated and will be removed after 2021-01-0
1.
Instructions for updating:
Please use `model.predict()` instead.
WARNING:tensorflow:From C:\Anaconda\lib\site-packages\tensorflow\python\keras
\wrappers\scikit_learn.py:241: Sequential.predict_classes (from tensorflow.py
thon.keras.engine.sequential) is deprecated and will be removed after 2021-01
-01.
Instructions for updating:
Please use instead:* `np.argmax(model.predict(x), axis=-1)`,   if your model
does multi-class classification   (e.g. if it uses a `softmax` last-layer act
ivation).* `(model.predict(x) > 0.5).astype("int32")`,   if your model does b
inary classification   (e.g. if it uses a `sigmoid` last-layer activation).
```

In [63]:
```
nn_roc_auc = roc_auc_score(y_test, nn_y_pred_prob).round(4)
nn_accuracy=accuracy_score(y_test, nn_y_pred).round(4)
nn_f1score = f1_score(y_test, nn_y_pred, average='binary')
nn_precision = precision_score(y_test, nn_y_pred, labels=[1,2], average='micr
o')
nn_recall = recall_score(y_test, nn_y_pred, labels=[1,2], average='micro')

print("nn_accuracy_score:%.3f, nn_preci_score:%.3f, nn_recall_score:%.3f, nn_f
1_score:%.3f, nn_auc:%.3f"
%(nn_accuracy, nn_precision, nn_recall, nn_f1score, nn_roc_auc))
```

```
nn_accuracy_score:0.752, nn_preci_score:0.615, nn_recall_score:0.375, nn_f1_s
core:0.466, nn_auc:0.757
```

In [64]:
```
#Data for ROC plot
nn_fpr, nn_tpr, nn_threasholds=roc_curve(y_test, nn_y_pred_prob)
```

In [65]:
```
##############################################
#SVM
##############################################
```

In [66]:
```
from sklearn.model_selection import GridSearchCV
from sklearn import svm
from sklearn.svm import SVC
from sklearn.metrics import classification_report
import warnings
warnings.filterwarnings('ignore')

grid_params = [{'kernel': ['rbf'], 'gamma': [1e-3, 1e-4],'C': [1, 10, 100, 100
0]},
              {'kernel': ['linear'], 'C': [1, 10, 100, 1000]},
              {'kernel': ['poly'], 'C': [1, 10, 100, 1000]},
              {'kernel': ['rbf'], 'C': [1, 10, 100, 1000]},
              {'kernel': ['sigmoid'], 'C': [1, 10, 100, 1000]}]

clf = GridSearchCV(svm.SVC(probability=True), grid_params,verbose=1, cv=10, n_
jobs=-1)
```

In [67]:
```
svmbest_clf=clf.fit(x_train, y_train)
```

```
Fitting 10 folds for each of 24 candidates, totalling 240 fits

[Parallel(n_jobs=-1)]: Using backend LokyBackend with 4 concurrent workers.
[Parallel(n_jobs=-1)]: Done  76 tasks      | elapsed:    1.8s
[Parallel(n_jobs=-1)]: Done 233 out of 240 | elapsed:  4.5min remaining:
8.0s
[Parallel(n_jobs=-1)]: Done 240 out of 240 | elapsed:  6.7min finished
```

In [68]:
```
svmbest_clf.best_params_
```

Out[68]:
```
{'C': 1000, 'gamma': 0.001, 'kernel': 'rbf'}
```

In [69]:
```
from sklearn.metrics import accuracy_score, precision_score, recall_score, f1_
score,roc_auc_score
svm_y_pred_prob=svmbest_clf.predict_proba(x_test)[:,1]
svm_y_pred=svmbest_clf.predict(x_test)
```

In [70]:
```
svm_roc_auc = roc_auc_score(y_test, svm_y_pred_prob).round(4)
svm_accuracy=accuracy_score(y_test, svm_y_pred).round(4)
svm_f1score = f1_score(y_test, svm_y_pred, average='binary')
svm_precision = precision_score(y_test, svm_y_pred, labels=[1,2], average='mic
ro')
svm_recall = recall_score(y_test, svm_y_pred, labels=[1,2], average='micro')

print("svm_accuracy_score:%.3f, svm_preci_score:%.3f, svm_recall_score:%.3f, s
vm_f1_score:%.3f, svm_auc:%.3f"
%(svm_accuracy, svm_precision, svm_recall, svm_f1score, svm_roc_auc))
```

```
svm_accuracy_score:0.748, svm_preci_score:0.654, svm_recall_score:0.266, svm_
f1_score:0.378, svm_auc:0.711
```

In [71]:
```
#Data for ROC plot
svm_fpr, svm_tpr, svm_threasholds=roc_curve(y_test, svm_y_pred_prob)
```

In [72]:
```
###############################################
#Naive Bayes
###############################################
from sklearn.naive_bayes import GaussianNB
nb = GaussianNB()
nb_y_pred = cross_val_predict(nb, x_test, y_test, cv=10)
nb_y_pred_prob=cross_val_predict(nb, x_test, y_test, cv=10,method='predict_pro
ba')

nb_roc_auc = roc_auc_score(y_test, nb_y_pred_prob[:,1]).round(4)
nb_accuracy=accuracy_score(y_test, nb_y_pred).round(4)
nb_f1score = f1_score(y_test, nb_y_pred, average='binary')
nb_precision = precision_score(y_test, nb_y_pred, labels=[1,2], average='micr
o')
nb_recall = recall_score(y_test, nb_y_pred, labels=[1,2], average='micro')

print("nb_accuracy_score:%.3f, nb_preci_score:%.3f, nb_recall_score:%.3f, nb_f
1_score:%.3f, nb_auc:%.3f"
%(nb_accuracy, nb_precision, nb_recall, nb_f1score, nb_roc_auc))
```

```
nb_accuracy_score:0.734, nb_preci_score:0.540, nb_recall_score:0.531, nb_f1_s
core:0.535, nb_auc:0.704
```

In [73]:
```
#Data for ROC plot
nb_fpr, nb_tpr, nb_threasholds=roc_curve(y_test, nb_y_pred_prob[:,1])
```

In [74]:
```
###############################################
#Unpenalised LR
###############################################
```

In [75]:
```
from sklearn.model_selection import cross_val_score, cross_val_predict
LR=LogisticRegression(penalty='none',max_iter=1000,warm_start=True).fit(x_test
, y_test)
LR_y_pred = cross_val_predict(LR, x_test, y_test, cv=10)
LR_y_pred_prob=cross_val_predict(LR, x_test, y_test, cv=10,method='predict_pro
ba')
LR_roc_auc = roc_auc_score(y_test, LR_y_pred_prob[:,1]).round(4)
LR_accuracy=accuracy_score(y_test, LR_y_pred).round(4)
LR_f1score = f1_score(y_test, LR_y_pred, average='binary')
LR_precision = precision_score(y_test, LR_y_pred, labels=[1,2], average='micr
o')
LR_recall = recall_score(y_test, LR_y_pred, labels=[1,2], average='micro')

print("LR_accuracy_score:%.3f, LR_preci_score:%.3f, LR_recall_score:%.3f, LR_f
1_score:%.3f, LR_auc:%.3f"
%(LR_accuracy, LR_precision, LR_recall, LR_f1score, LR_roc_auc))
```

```
LR_accuracy_score:0.725, LR_preci_score:0.537, LR_recall_score:0.344, LR_f1_s
core:0.419, LR_auc:0.690
```

In [76]:
```
#Data for ROC plot
LR_fpr, LR_tpr, LR_threasholds=roc_curve(y_test, LR_y_pred_prob[:,1])
```

In [77]:
```
################################
#ROC plot
################################
```

In [78]:
```
lr_fpr, lr_tpr, lr_threasholds=roc_curve(y_test, lr_y_pred_prob)
dt_fpr, dt_tpr, dt_threasholds=roc_curve(y_test, dt_y_pred_prob)
rf_fpr, rf_tpr, rf_threasholds=roc_curve(y_test, rf_y_pred_prob)
xgb_fpr, xgb_tpr, xgb_threasholds=roc_curve(y_test, xgb_y_pred_prob)
knn_fpr, knn_tpr, knn_threasholds=roc_curve(y_test, knn_y_pred_prob)
nn_fpr, nn_tpr, nn_threasholds=roc_curve(y_test, nn_y_pred_prob)
svm_fpr, svm_tpr, svm_threasholds=roc_curve(y_test, svm_y_pred_prob)
LR_fpr, LR_tpr, LR_threasholds=roc_curve(y_test, LR_y_pred_prob[:,1])
nb_fpr, nb_tpr, nb_threasholds=roc_curve(y_test, nb_y_pred_prob[:,1])

plt.plot(dt_fpr, dt_tpr,c='g', lw=1, label=u' dt,AUC=%.3f'% dt_roc_auc)
plt.plot(knn_fpr, knn_tpr,c='darkorange', lw=1, label=u' knn,AUC=%.3f'% knn_ro
c_auc)
plt.plot(LR_fpr, LR_tpr,c='pink', lw=1, label=u' nplr,AUC=%.3f'% LR_roc_auc)
plt.plot(svm_fpr, svm_tpr,c='grey', lw=1, label=u' svm,AUC=%.3f'% svm_roc_auc)
plt.plot(nb_fpr, nb_tpr,c='b', lw=1, label=u' nb,AUC=%.3f'% nb_roc_auc)
plt.plot(lr_fpr, lr_tpr,c='r', lw=1, label=u' plr,AUC=%.3f'% lr_roc_auc)
plt.plot(nn_fpr, nn_tpr,c='darkblue', lw=1, label=u' nn,AUC=%.3f'% nn_roc_auc)
plt.plot(rf_fpr, rf_tpr,c='y', lw=1, label=u' rf,AUC=%.3f'% rf_roc_auc)
plt.plot(xgb_fpr, xgb_tpr,c='b', lw=1, label=u' xgb,AUC=%.3f'% xgb_roc_auc)

plt.plot ((0, 1), (0, 1), c='0', lw=1, ls='--')
plt.xlim(-0.001, 1.001)
plt.ylim(-0.001, 1.001)
plt.xticks(np.arange(0, 1.1, 0.1))
plt.yticks(np.arange(0, 1.1, 0.1))
plt.xlabel('False Positive Rate', fontsize= 16)
plt.ylabel('True Positive Rate', fontsize=16)
plt.grid (b=True, ls='-')
plt.legend(loc='lower right', fancybox=True, framealpha=0.8, fontsize=12)
plt.title(u'', fontsize=18)
plt.savefig('ROC Test Data2.png', dpi=1200)
```

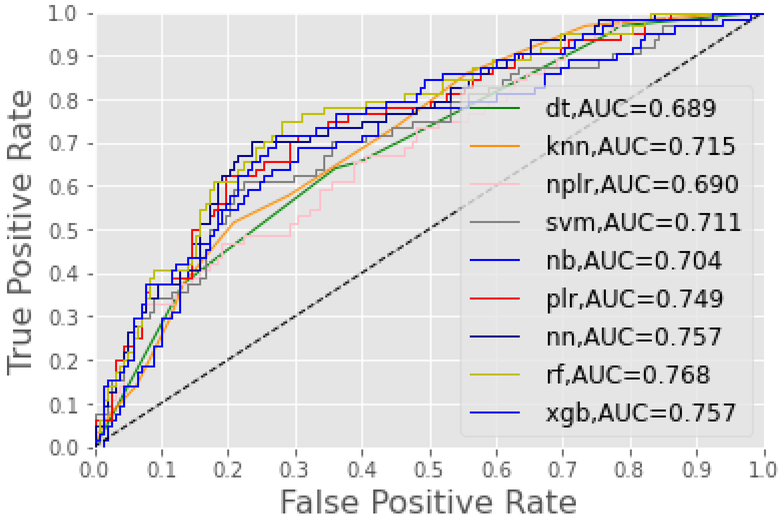

In [79]:
```
#############################################
#Combine roc plots for datasets 1-4
#############################################
roc1=pd.read_excel('roc1.xlsx', index_col=0)
auc1=pd.read_excel('auc1.xlsx', index_col=0)
roc2=pd.read_excel('roc2.xlsx', index_col=0)
auc2=pd.read_excel('auc2.xlsx', index_col=0)
roc3=pd.read_excel('roc3.xlsx', index_col=0)
auc3=pd.read_excel('auc3.xlsx', index_col=0)
```

In [80]:

```
#fig, axs = plt.subplots(ncols=2, nrows=2)
fig, axs = plt.subplots(2,2,figsize=(14,14))
plt.style.use('ggplot')

#ax1.set_xticks(numpy.arange(0, 0.14, 0.02))
plt.subplot(2, 2, 1)
ax1=plt.plot(roc1['dt_fpr'], roc1['dt_tpr'],c='g', lw=1, label=u' dt,AUC=%.3f'
% auc1['dt_roc_auc'])
plt.plot(roc1['knn_fpr'], roc1['knn_tpr'],c='darkorange', lw=1, label=u' knn,A
UC=%.3f'% auc1['knn_roc_auc'])
plt.plot(roc1['LR_fpr'], roc1['LR_tpr'],c='pink', lw=1, label=u' lr,AUC=%.3f'%
auc1['LR_roc_auc'])
plt.plot(roc1['nb_fpr'], roc1['nb_tpr'],c='b', lw=1, label=u' nb,AUC=%.3f'% au
c1['nb_roc_auc'])
plt.plot(roc1['rf_fpr'], roc1['rf_tpr'],c='y', lw=1, label=u' rf,AUC=%.3f'% au
c1['rf_roc_auc'])
plt.plot(roc1['svm_fpr'], roc1['svm_tpr'],c='grey', lw=1, label=u' svm,AUC=%.3
f'% auc1['svm_roc_auc'])
plt.plot(roc1['lr_fpr'], roc1['lr_tpr'],c='r', lw=1, label=u' ridge,AUC=%.3f'%
auc1['lr_roc_auc'])
plt.plot(roc1['nn_fpr'], roc1['nn_tpr'],c='darkblue', lw=1, label=u' nn,AUC=%.
3f'% auc1['nn_roc_auc'])
plt.plot(roc1['xgb_fpr'], roc1['xgb_tpr'],c='b', lw=1, label=u' xgb,AUC=%.3f'%
auc1['xgb_roc_auc'])
plt.plot ((0, 1), (0, 1), c='0', lw=1, ls='--')
plt.xlim(-0.001, 1.001)
plt.ylim(-0.001, 1.001)
plt.xticks(np.arange(0, 1.1, 0.1))
plt.yticks(np.arange(0, 1.1, 0.1))
plt.xlabel('False Positive Rate', fontsize= 16)
plt.ylabel('True Positive Rate', fontsize=16)
plt.grid (b=True, ls='-')
plt.legend(loc='lower right', fancybox=True, framealpha=0.8, fontsize=12)
plt.title(u'ROC Test Feature-set 1', fontsize=18)

plt.subplot(2, 2, 2)
ax1=plt.plot(roc2['dt_fpr'], roc2['dt_tpr'],c='g', lw=1, label=u' dt,AUC=%.3f'
% auc2['dt_roc_auc'])
plt.plot(roc2['knn_fpr'], roc2['knn_tpr'],c='darkorange', lw=1, label=u' knn,A
UC=%.3f'% auc2['knn_roc_auc'])
plt.plot(roc2['LR_fpr'], roc2['LR_tpr'],c='pink', lw=1, label=u' lr,AUC=%.3f'%
auc2['LR_roc_auc'])
plt.plot(roc2['nb_fpr'], roc2['nb_tpr'],c='b', lw=1, label=u' nb,AUC=%.3f'% au
c2['nb_roc_auc'])
plt.plot(roc2['rf_fpr'], roc2['rf_tpr'],c='y', lw=1, label=u' rf,AUC=%.3f'% au
c2['rf_roc_auc'])
plt.plot(roc2['svm_fpr'], roc2['svm_tpr'],c='grey', lw=1, label=u' svm,AUC=%.3
f'% auc2['svm_roc_auc'])
plt.plot(roc2['lr_fpr'], roc2['lr_tpr'],c='r', lw=1, label=u' ridge,AUC=%.3f'%
auc2['lr_roc_auc'])
plt.plot(roc2['nn_fpr'], roc2['nn_tpr'],c='darkblue', lw=1, label=u' nn,AUC=%.
3f'% auc2['nn_roc_auc'])
plt.plot(roc2['xgb_fpr'], roc2['xgb_tpr'],c='b', lw=1, label=u' xgb,AUC=%.3f'%
auc2['xgb_roc_auc'])
plt.plot ((0, 1), (0, 1), c='0', lw=1, ls='--')
plt.xlim(-0.001, 1.001)
```

```
plt.ylim(-0.001, 1.001)
plt.xticks(np.arange(0, 1.1, 0.1))
plt.yticks(np.arange(0, 1.1, 0.1))
plt.xlabel('False Positive Rate', fontsize= 16)
plt.ylabel('True Positive Rate', fontsize=16)
plt.grid (b=True, ls='-')
plt.legend(loc='lower right', fancybox=True, framealpha=0.8, fontsize=12)
plt.title(u'ROC Test Feature-set 2', fontsize=18)

plt.subplot(2, 2, 3)
ax1=plt.plot(roc3['dt_fpr'], roc3['dt_tpr'],c='g', lw=1, label=u' dt,AUC=%.3f'
% auc3['dt_roc_auc'])
plt.plot(roc3['knn_fpr'], roc3['knn_tpr'],c='darkorange', lw=1, label=u' knn,A
UC=%.3f'% auc3['knn_roc_auc'])
plt.plot(roc3['LR_fpr'], roc3['LR_tpr'],c='pink', lw=1, label=u' lr,AUC=%.3f'%
auc3['LR_roc_auc'])
plt.plot(roc3['nb_fpr'], roc3['nb_tpr'],c='b', lw=1, label=u' nb,AUC=%.3f'% au
c3['nb_roc_auc'])
plt.plot(roc3['rf_fpr'], roc3['rf_tpr'],c='y', lw=1, label=u' rf,AUC=%.3f'% au
c3['rf_roc_auc'])
plt.plot(roc3['svm_fpr'], roc3['svm_tpr'],c='grey', lw=1, label=u' svm,AUC=%.3
f'% auc3['svm_roc_auc'])
plt.plot(roc3['lr_fpr'], roc3['lr_tpr'],c='r', lw=1, label=u' ridge,AUC=%.3f'%
auc3['lr_roc_auc'])
plt.plot(roc3['nn_fpr'], roc3['nn_tpr'],c='darkblue', lw=1, label=u' nn,AUC=%.
3f'% auc3['nn_roc_auc'])
plt.plot(roc3['xgb_fpr'], roc3['xgb_tpr'],c='b', lw=1, label=u' xgb,AUC=%.3f'%
auc3['xgb_roc_auc'])
plt.plot ((0, 1), (0, 1), c='0', lw=1, ls='--')
plt.xlim(-0.001, 1.001)
plt.ylim(-0.001, 1.001)
plt.xticks(np.arange(0, 1.1, 0.1))
plt.yticks(np.arange(0, 1.1, 0.1))
plt.xlabel('False Positive Rate', fontsize= 16)
plt.ylabel('True Positive Rate', fontsize=16)
plt.grid (b=True, ls='-')
plt.legend(loc='lower right', fancybox=True, framealpha=0.8, fontsize=12)
plt.title(u'ROC Test Feature-set 3', fontsize=18)

plt.subplot(2, 2, 4)
plt.plot(dt_fpr, dt_tpr,c='g', lw=1, label=u' dt,AUC=%.3f'% dt_roc_auc)

plt.plot(LR_fpr, LR_tpr,c='pink', lw=1, label=u' lr,AUC=%.3f'% LR_roc_auc)
plt.plot(svm_fpr, svm_tpr,c='grey', lw=1, label=u' svm,AUC=%.3f'% svm_roc_auc)
plt.plot(nb_fpr, nb_tpr,c='b', lw=1, label=u' nb,AUC=%.3f'% nb_roc_auc)
plt.plot(knn_fpr, knn_tpr,c='darkorange', lw=1, label=u' knn,AUC=%.3f'% knn_ro
c_auc)
plt.plot(lr_fpr, lr_tpr,c='r', lw=1, label=u' ridge,AUC=%.3f'% lr_roc_auc)
plt.plot(rf_fpr, rf_tpr,c='y', lw=1, label=u' rf,AUC=%.3f'% rf_roc_auc)
plt.plot(nn_fpr, nn_tpr,c='darkblue', lw=1, label=u' nn,AUC=%.3f'% nn_roc_auc)
plt.plot(xgb_fpr, xgb_tpr,c='b', lw=1, label=u' xgb,AUC=%.3f'% xgb_roc_auc)

plt.plot ((0, 1), (0, 1), c='0', lw=1, ls='--')
plt.xlim(-0.001, 1.001)
plt.ylim(-0.001, 1.001)
plt.xticks(np.arange(0, 1.1, 0.1))
plt.yticks(np.arange(0, 1.1, 0.1))
```

```
plt.xlabel('False Positive Rate', fontsize= 16)
plt.ylabel('True Positive Rate', fontsize=16)
plt.grid (b=True, ls='-')
plt.legend(loc='lower right', fancybox=True, framealpha=0.8, fontsize=12)
plt.title(u'ROC Test Feature-set 4', fontsize=18)

plt.savefig('ROC Test Data1+Data2_Data3+data4.png', dpi=1200)
plt.show()
```

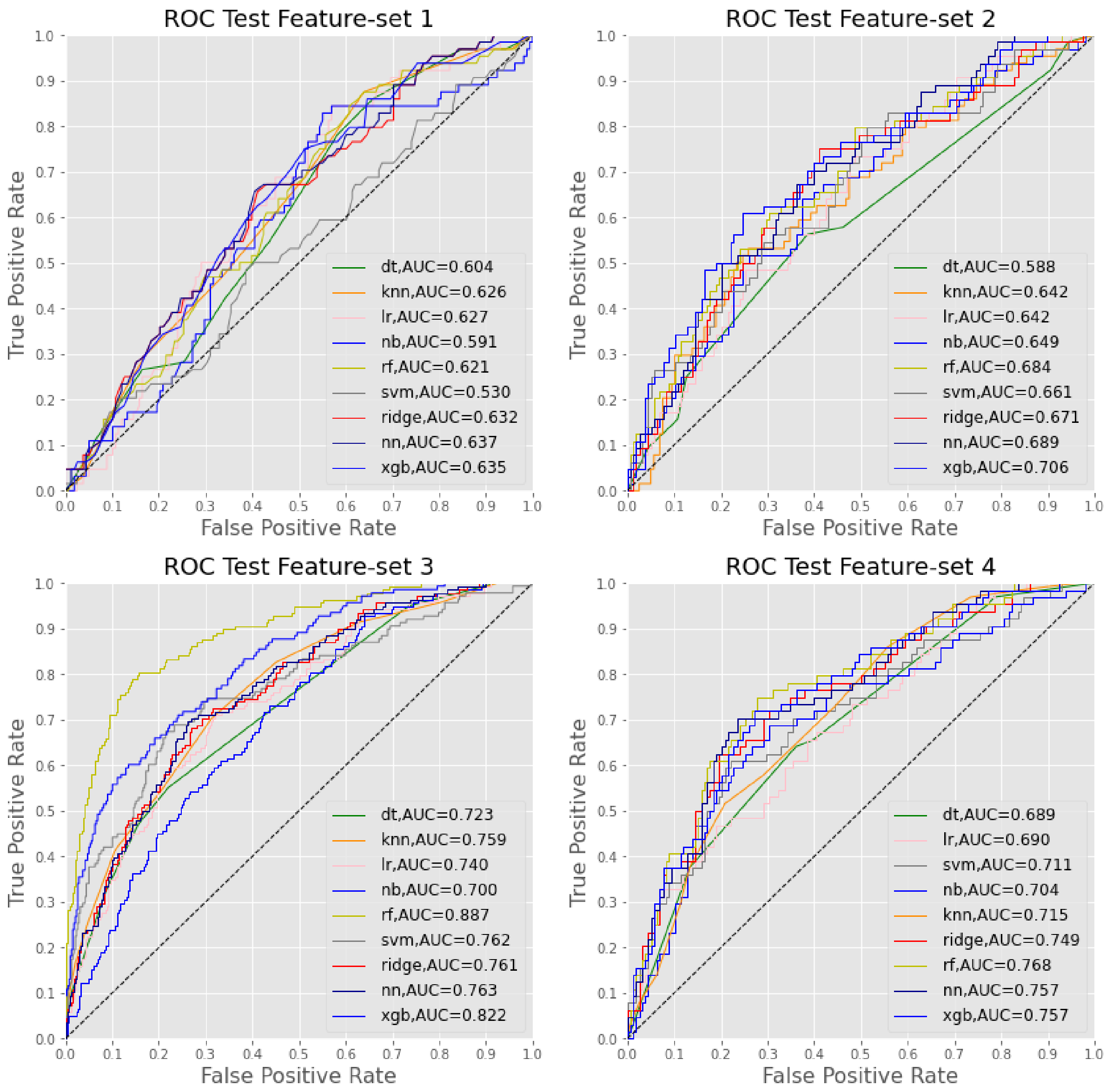

In [ ]:

In [82]:

```
####################################
#Precision-Recall Curve
####################################
```

In [83]:

```
# calculate precision-recall curve
from sklearn.metrics import precision_recall_curve
precision, recall, thresholds = precision_recall_curve(y_test, xgb_y_pred_prob
)
```

In [84]:
```
# calculate precision-recall AUC
from sklearn.metrics import auc
auc = auc(recall, precision)
```

In [85]:

```
#4 plots for XGBoost together
plt.figure(figsize=(10, 10))
plt.style.use('ggplot')

plt.subplot(221)
plt.hist(xgb_y_pred_prob, range=(0, 1), bins=15, label=None,
             histtype="bar", lw=2,facecolor='gray',rwidth=0.8)
plt.xlabel("Mean predicted value", fontsize=12)
plt.ylabel("Count", fontsize=12)
plt.legend(loc="upper center", ncol=1)
plt.title(u'XGBoost Predicted risk', fontsize=18)

plt.subplot(222)
plt.plot([0, 1], [0, 1], "k:", label="Perfectly calibrated")
xgb_y_pred_prob = \
            (xgb_y_pred_prob - xgb_y_pred_prob.min()) / (xgb_y_pred_prob.max()
- xgb_y_pred_prob.min())
fraction_of_positives, mean_predicted_value = \
        calibration_curve(y_test, xgb_y_pred_prob, n_bins=10)
plt.plot(mean_predicted_value, fraction_of_positives, "s-",
             label=None)
plt.ylabel("Fraction of positives")
plt.ylim([-0.05, 1.05])
plt.legend(loc="lower right")
plt.grid (b=True, ls='-')
plt.title('XGBoost Calibration', fontsize=18)
plt.xlabel("Mean predicted value", fontsize=12)

plt.subplot(223)
no_skill = len(y_test[y_test==1]) / len(y_test)
plt.plot([0, 1], [no_skill, no_skill], linestyle='--', label='No Skill',c='gra
y')
plt.plot(recall, precision, marker='.', label=None)
plt.xlabel('Recall', fontsize=12)
plt.ylabel('Precision', fontsize=12)
plt.grid (b=True, ls='-')
plt.legend()
plt.title(u'XGBoost Precision-Recall', fontsize=18)
plt.ylim([-0.05, 1.05])

plt.subplot(224)
xgb_fpr, xgb_tpr, xgb_threasholds=roc_curve(y_test, xgb_y_pred_prob)
plt.plot(xgb_fpr, xgb_tpr, lw=2, label=u' AUC=%.3f'% xgb_roc_auc)
plt.plot ((0, 1), (0, 1), c='gray', lw=2, ls='--')
plt.xlim(-0.001, 1.001)
plt.ylim(-0.001, 1.001)
plt.xticks(np.arange(0, 1.1, 0.1))
plt.yticks(np.arange(0, 1.1, 0.1))
plt.xlabel('False Positive Rate', fontsize= 12)
plt.ylabel('True Positive Rate', fontsize=12)
plt.grid (b=True, ls='-')
plt.legend(loc='lower right', fancybox=True, framealpha=0.8, fontsize=12)
plt.title(u'XGBoost AUROC', fontsize=18)

plt.subplots_adjust(top=0.92, bottom=0.08, left=0.10, right=0.95, hspace=0.25,
                    wspace=0.35)
```

```
plt.savefig('XGBoostAccPlts.png', dpi=1200)
plt.show()
```

No handles with labels found to put in legend.

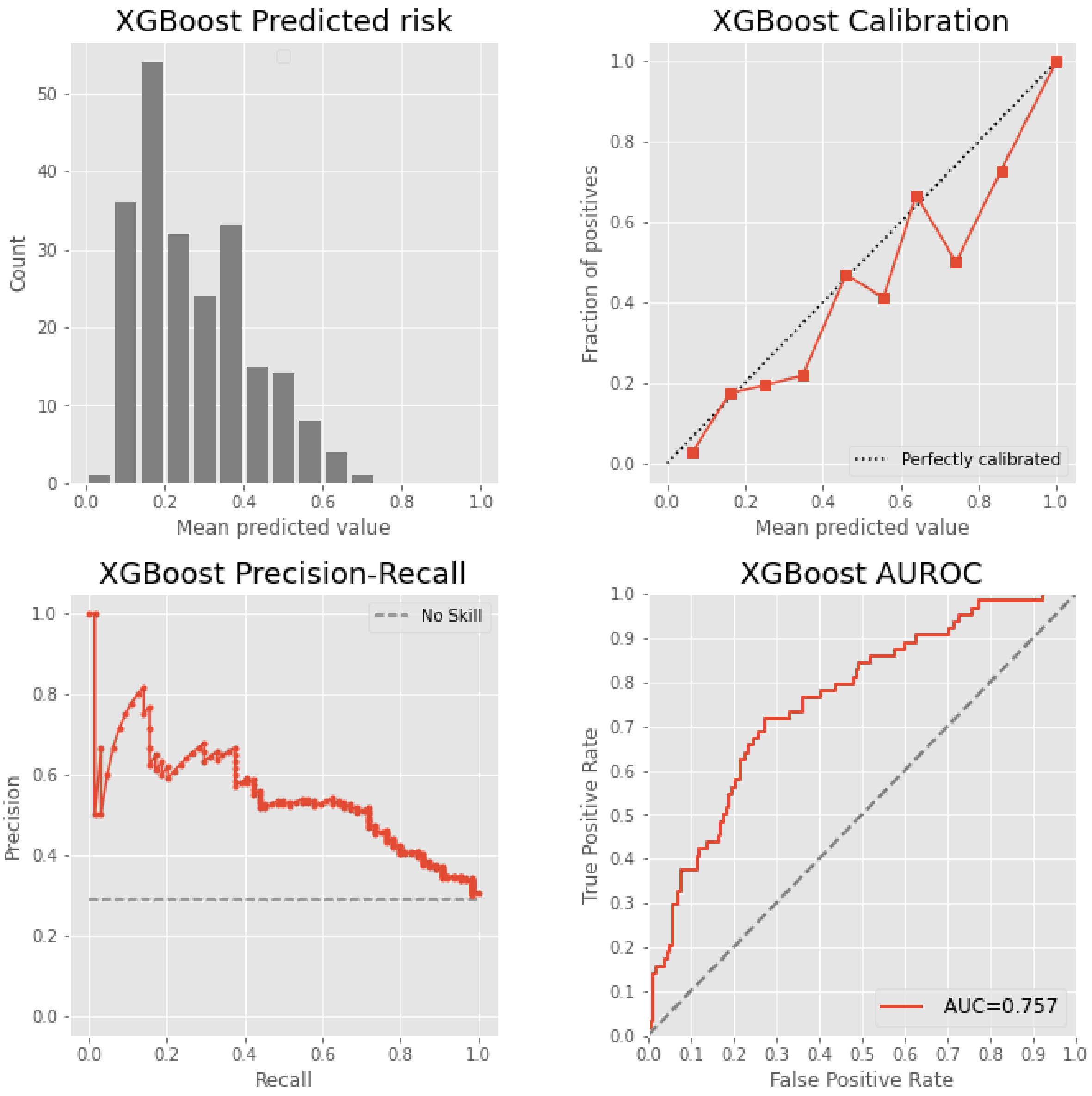

Supplement: Multimedia Appendix 7 [file jmir_v23i6e26139_app7.pdf]
