# Supplementary material for: Use of Multiprognostic Index Domain Scores, Clinical Data, and Machine Learning to Improve 12-Month Mortality Risk Prediction in Older Hospitalized Patients: Prospective Cohort Study
Source: J Med Internet Res. 2021 Jun 21;23(6):e26139. doi: 10.2196/26139 (PMC8277374; doi:10.2196/26139)
Supplement: Multimedia Appendix 8 [file jmir_v23i6e26139_app8.docx]

**Supplementary Table 1:** Diagnostic accuracy for LR-MLE and the 9 ML algorithms using feature-sets 1 to 4 with the training dataset.

|  |  |  | **AUROC** |  |  |
| --- | --- | --- | --- | --- | --- |
|  | **Feature-set 1** | **Feature-set 2** | **Feature-set 3** | **Feature-set 4** | **Mean AUC** |
| LR MLE | 0.640 | 0.708 | 0.761 | 0.778 | 0.722 |
| ML algorithms |  |  |  |  |  |
| RF | 0.724 | 0.933 | 0.887 | 0.956 | 0.875 |
| XGB | 0.682 | 0.891 | 0.822 | 0.877 | 0.818 |
| KNN | 0.682 | 1.000 | 0.759 | 0.761 | 0.800 |
| SVM | 0.632 | 0.750 | 0.762 | 0.855 | 0.750 |
| NN | 0.644 | 0.751 | 0.763 | 0.810 | 0.742 |
| DT | 0.679 | 0.714 | 0.723 | 0.754 | 0.718 |
| Ridge | 0.640 | 0.704 | 0.761 | 0.772 | 0.719 |
| LR | 0.628 | 0.670 | 0.740 | 0.730 | 0.692 |
| NB | 0.623 | 0.658 | 0.700 | 0.700 | 0.670 |

AUROC=Area-under-receiver-operating-curve

Feature-set 1=MPI categories, age, gender (n=5 features)

Feature-set 2=MPI categories, age, gender, BMI, Anticholinergic risk score, Lab data (n=15 features)

Feature-set 3=MPI Domains, age, gender (n=10 features)

Feature-set 4=MPI Domains, age, gender, BMI, Anticholinergic risk score, Lab data (n=20 features)

Lab data=serum albumin, Na, Hgb, CRP, Cr, Urea, Urea/Cr ratio, eGFR.

LR MLE=Logistic regression using Maximum likelihood Estimation

XGB=Extreme gradient boosting; NN=Neural Network; Ridge=Ridge regression; RF=Random Forest; KNN=K Nearest Neighbours; SVM=Support Vector Machine; NB=Naïve Bayes; LR=Non-penalised Logistic Regression; DT=Decision Tree.
