# Supplementary material for: Use of Multiprognostic Index Domain Scores, Clinical Data, and Machine Learning to Improve 12-Month Mortality Risk Prediction in Older Hospitalized Patients: Prospective Cohort Study
Source: J Med Internet Res. 2021 Jun 21;23(6):e26139. doi: 10.2196/26139 (PMC8277374; doi:10.2196/26139)
Supplement: Multimedia Appendix 9 [file jmir_v23i6e26139_app9.docx]

**Supplementary Table 2:** Precision, recall, accuracy and F1-score for LR-MLE and the 9 ML algorithms using feature-set 4 with the training dataset.

|  | **Accuracy** | **Precision** | **Recall** | **F1-score** |
| --- | --- | --- | --- | --- |
| LR MLE | 0.748 | 0.611 | 0.344 | 0.440 |
| ML algorithms |  |  |  |  |
| RF | 0.739 | 0.650 | 0.203 | 0.310 |
| XGB | 0.739 | 0.625 | 0.234 | 0.341 |
| KNN | 0.707 | 0.474 | 0.141 | 0.217 |
| SVM | 0.748 | 0.654 | 0.266 | 0.711 |
| NN | 0.752 | 0.615 | 0.375 | 0.466 |
| DT | 0.712 | 0.500 | 0.031 | 0.059 |
| Ridge | 0.743 | 0.640 | 0.250 | 0.360 |
| LR | 0.725 | 0.537 | 0.344 | 0.419 |
| NB | 0.734 | 0.540 | 0.531 | 0.535 |

Feature-set 4=MPI Domains, age, gender, BMI, Anticholinergic risk score, Lab data (n=20 features)

Lab data=serum albumin, Na, Hgb, CRP, Cr, Urea, Urea/Cr ratio, eGFR.

LR MLE=Logistic regression using Maximum likelihood Estimation

XGB=Extreme gradient boosting; NN=Neural Network; Ridge=Ridge regression; RF=Random Forest; KNN=K Nearest Neighbours; SVM=Support Vector Machine; NB=Naïve Bayes; LR=Non-penalised Logistic Regression; DT=Decision Tree.
