# Supplementary figures and images for: Use of Multiprognostic Index Domain Scores, Clinical Data, and Machine Learning to Improve 12-Month Mortality Risk Prediction in Older Hospitalized Patients: Prospective Cohort Study
Source: J Med Internet Res. 2021 Jun 21;23(6):e26139. doi: 10.2196/26139 (PMC8277374; doi:10.2196/26139)

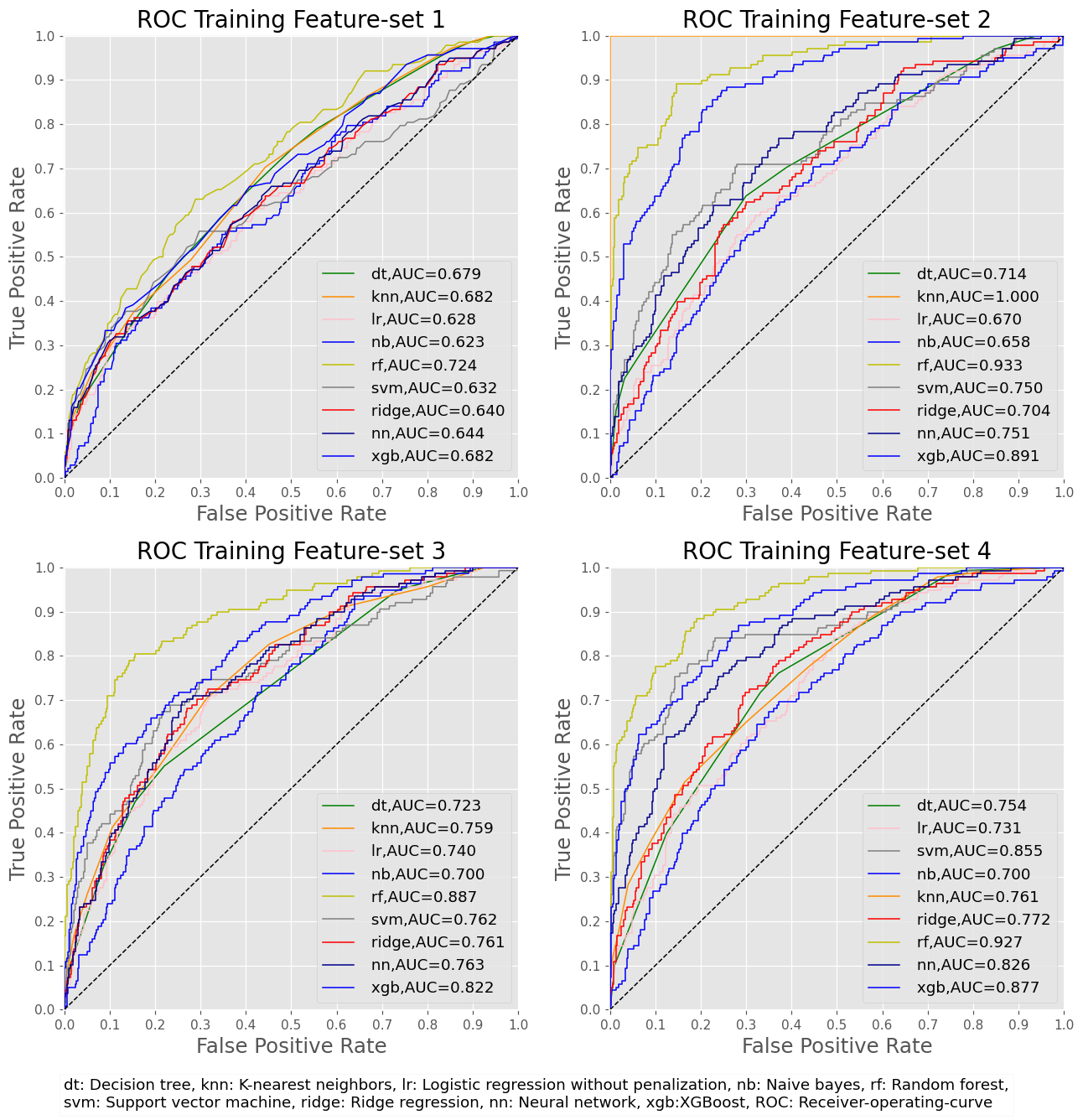

Supplement: Multimedia Appendix 10 [file jmir_v23i6e26139_app10.png]

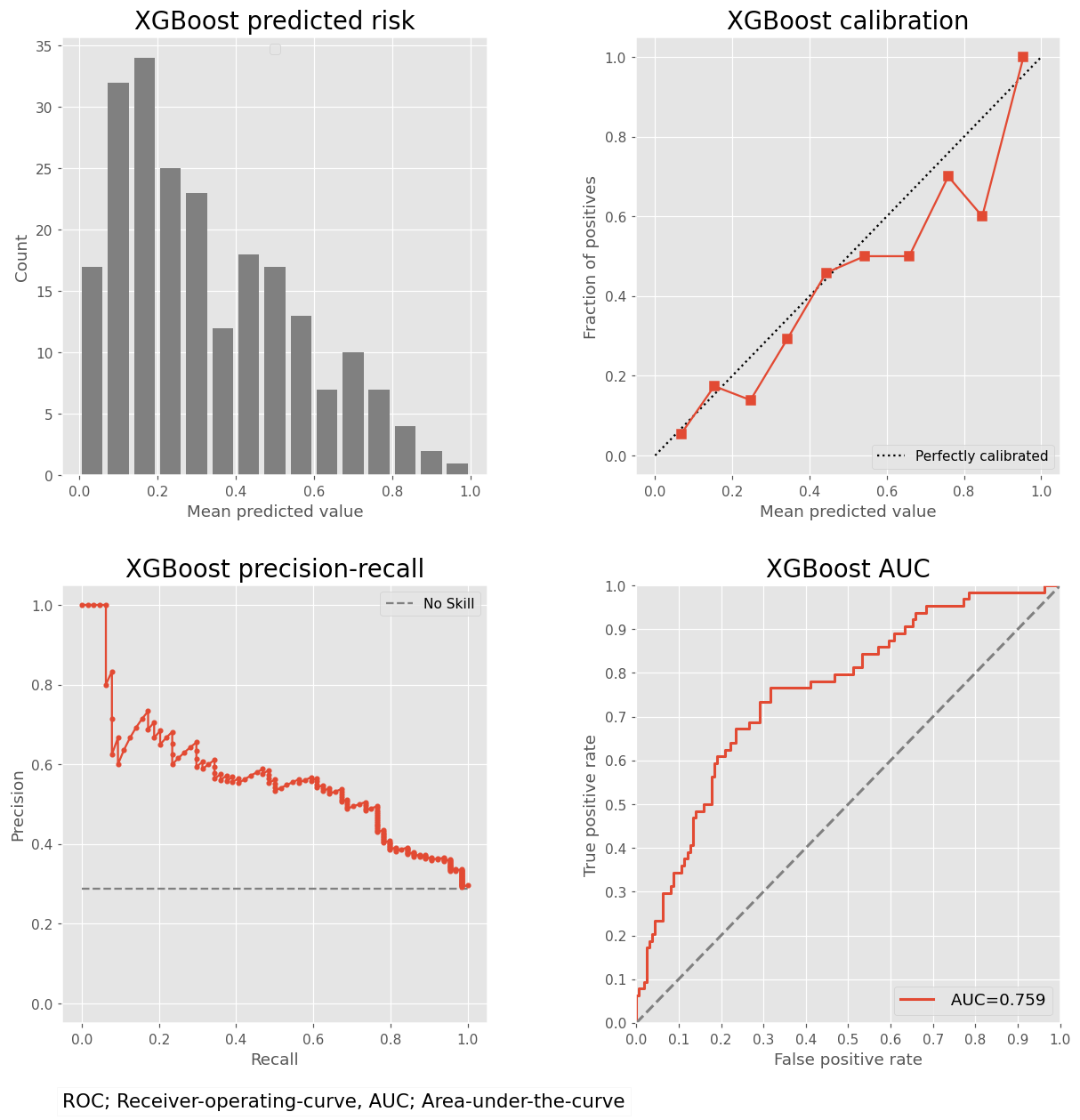

Supplement: Multimedia Appendix 11 [file jmir_v23i6e26139_app11.png]
